# Subtyping of circulating exosome-bound amyloid β reflects brain plaque deposition

**DOI:** 10.1038/s41467-019-09030-2

**Published:** 2019-03-08

**Authors:** Carine Z. J. Lim, Yan Zhang, Yu Chen, Haitao Zhao, Mary C. Stephenson, Nicholas R. Y. Ho, Yuan Chen, Jaehoon Chung, Anthonin Reilhac, Tze Ping Loh, Christopher L. H. Chen, Huilin Shao

**Affiliations:** 1 Department of Biomedical Engineering, Faculty of Engineering, National University of Singapore, Singapore, 117583 Singapore; 2 Biomedical Institute for Global Health Research and Technology, National University of Singapore, Singapore, 117599 Singapore; 3 Institute of Microelectronics, Agency for Science, Technology and Research, Singapore, 138634 Singapore; 4 Clinical Imaging Research Center, National University of Singapore, Singapore, 117599 Singapore; 5 Institute of Molecular and Cell Biology, Agency for Science, Technology and Research, Singapore, 138673 Singapore; 6 Department of Laboratory Medicine, National University Hospital, Singapore, 119074 Singapore; 7 Memory Ageing and Cognition Center, National University Hospital, Singapore, 117599 Singapore; 8 Department of Pharmacology, Yong Loo Lin School of Medicine, National University of Singapore, Singapore, 117600 Singapore; 9 Department of Surgery, Yong Loo Lin School of Medicine, National University of Singapore, Singapore, 119228 Singapore

## Abstract

Despite intense interests in developing blood measurements of Alzheimer’s disease (AD), the progress has been confounded by limited sensitivity and poor correlation to brain pathology. Here, we present a dedicated analytical platform for measuring different populations of circulating amyloid β (Aβ) proteins – exosome-bound vs. unbound – directly from blood. The technology, termed **a**mplified **p**lasmonic **ex**osome (APEX), leverages in situ enzymatic conversion of localized optical deposits and double-layered plasmonic nanostructures to enable sensitive, multiplexed population analysis. It demonstrates superior sensitivity (~200 exosomes), and enables diverse target co-localization in exosomes. Employing the platform, we find that prefibrillar Aβ aggregates preferentially bind with exosomes. We thus define a population of Aβ as exosome-bound (Aβ42+ CD63+) and measure its abundance directly from AD and control blood samples. As compared to the unbound or total circulating Aβ, the exosome-bound Aβ measurement could better reflect PET imaging of brain amyloid plaques and differentiate various clinical groups.

## Abstract (web summary)

Detecting Alzheimer’s disease from blood samples is challenging because amyloid β blood levels are lower than the ELISA detection limit. Here the authors capture amyloid β bound to circulating exosomes on a plasmonic nanosensor, followed by enzymatic amplification to improve detection sensitivity.

## Introduction

Alzheimer’s disease (AD) is the public health crisis of the 21^st^ century. The most common form of severe dementia, AD is characterized by a progressive loss of memory and cognitive functions. However, long before these full-blown clinical symptoms appear, AD molecular hallmarks, notably extracellular amyloid β (Aβ) plaques, may manifest and advance^1^. Due to the complex and progressive neuropathology, early detection and intervention are thought to be essential to the success of disease-modifying therapies^2^. Current AD diagnosis and disease monitoring, however, are subjective and late-stage. They are achieved through clinical and neuropsychological assessments using published criteria^3,4^. New molecular assays are being developed, including cerebrospinal fluid measurements^5^ and brain amyloid plaque imaging through positron emission tomography (PET)^6^; however, these tests face limitations as they either require invasive lumbar punctures or are too expensive for wider clinical adoption. As a result, there is an intense interest in developing blood measurements of AD^7,8^.

Despite recent progress, blood-based AD measurements are challenging. First, unlike that in cerebrospinal fluid, pathological AD molecules in the circulation demonstrate a much lower concentration^9^. Plasma Aβ levels tend to be near the lower limits of detection of conventional ELISA assays; this limitation could have contributed to several conflicting findings in published reports^10^ and necessitates extensive processing in new detection technologies^11,12^. Second, little correlation has been established between plasma Aβ analysis with brain plaque deposition, the earliest pathological hallmark of AD^1,13^. One possible reason for this could arise from the different measurement methodologies. PET imaging probes, commonly used to determine brain amyloid burden, preferentially measure insoluble, fibrillar deposits while current blood-based approaches measure total soluble Aβ in plasma^6,9^. However, this difference warrants a more fundamental question—if there are subpopulations of circulating Aβ proteins that could better reflect the fibrillar pathology in the brain.

Exosomes have recently emerged as an attractive circulating biomarker. Exosomes are nanoscale extracellular membrane vesicles (50–150 nm in diameter) actively secreted by cells into the circulation^14–18^. As a robust messenger, they abound in blood, carry reflective molecular cargos across biological barriers (e.g., the blood brain barrier)^19–22^ and facilitate diverse intercellular communication^23,24^. Recent studies have identified that exosomes may play a significant role in AD pathogenesis and progression; exosomes associate with pathological AD proteins^25,26^ while exosome markers are enriched in human brain amyloid plaques^27^. Capturing this exchange of exosome-bound information could thus present a transformative, blood-based opportunity to molecularly characterize AD.

Here we describe a highly sensitive analytical platform for multiplexed exosome population analysis directly from blood samples of AD patients. The system, termed amplified plasmonic exosome (APEX), leverages in situ enzymatic amplification of optical deposits and transmission surface plasmon resonance (SPR)^28–30^ to enable multiplexed population analysis. In comparison to published exosome platforms^17,31^, we advance the APEX assay, sensor design and fabrication to maximize its detection capabilities. Specifically, we establish a sensitive assay technology to enzymatically deposit localized optical products for SPR signal amplification and molecular co-localization. Insoluble optical deposits are locally formed only when multiple molecular targets are concurrently present in exosomes. To complement the APEX assay, we pattern size-matching plasmonic nanostructures in a coupled, double-layered photonic system for enhanced bidirectional SPR measurements. The developed technology not only shows superior detection sensitivity (~200 exosomes), but also enables diverse measurements of target co-localization in exosomes.

Employing the APEX platform, we measure different populations of circulating Aβ proteins (exosome-bound, unbound and total) from blood. We evaluate the association of exosomes from different cell origins with various structural forms of pathological Aβ proteins. We find that prefibrillar Aβ aggregates preferentially bind with exosomes and thus define a population of circulating amyloid as exosome-bound (Aβ42+ CD63+). Using blood samples of patients across the AD clinical spectrum as well as control subjects (healthy controls and clinical controls with other neurodegenerative and neurovascular diseases), we use the APEX platform to measure different populations of circulating Aβ and correlate these blood measurements to PET imaging of brain amyloid plaque load. We find that across all clinical groups tested, in comparison to the populations of unbound Aβ or total circulating Aβ in blood, the exosome-bound Aβ population could strongly reflect brain plaque deposition and distinguish the clinical groups.

## Results

### Amplified plasmonic analysis of exosome-bound Aβ

**Fig. 1:**
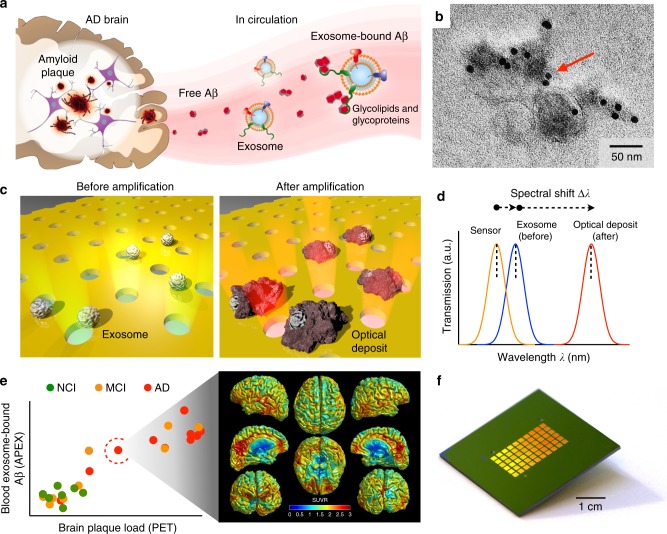
APEX platform for analysis of circulating exosome-bound Aβ. **a** Exosomes associate with Aβ proteins. Aβ proteins, the main component of amyloid plaques found in AD brain pathology, are released into the extracellular space. Exosomes are nanoscale extracellular membrane vesicles actively secreted by mammalian cells. Through their surface glycoproteins and glycolipids, exosomes can associate with the released Aβ proteins. **b** Transmission electron micrograph of exosome-bound Aβ. Exosomes derived from neuronal cells (SH-SY5Y) were treated with Aβ42 aggregates, and labeled with gold nanoparticles (10 nm) via a Aβ42-specific antibody. The nanoparticles appear as block dots (indicated by the red arrow). **c** APEX assay schematics. To enable sensitive profiling at the nanoscale, exosomes are first immuno-captured onto a plasmonic nanosensor (before amplification). Through an in situ enzymatic amplification, insoluble optical deposits are locally formed on the sensor-bound exosomes (after amplification). This deposition is spatially defined for molecular co-localization analysis, and changes the refractive index for SPR signal enhancement. To complement the enzymatic amplification, the nanosensor is back illuminated (away from the enzyme activity) to achieve analytical stability. The deposition causes a resultant red shift in the transmitted light through the nanosensor. **d** A representative schematic of changes in the transmission spectra with APEX amplification. Specific exosome binding (before) and subsequent amplification profiling (after) were monitored as transmission spectral shifts (Δλ) by the APEX platform. a.u., arbitrary unit. **e** Exosome-bound Aβ was measured using the APEX platform, in blood samples of patients with Alzheimer’s disease (AD), mild cognitive impairment (MCI), and controls with no cognitive impairment (NCI). The blood measurements were correlated to corresponding PET imaging of brain amyloid plaque deposition. **f** A photograph of the APEX microarray. Each sensor chip contains 6 × 10 sensing elements, made up of uniformly fabricated plasmonic lattice, for multiplexed measurements. See Supplementary Fig. 3–6 for sensor fabrication, characterization and design optimization, respectively

One of the earliest pathological hallmarks of AD is brain deposits of Aβ. These plaques are formed from the clustering of abnormal amyloid protein fragments, majorly the hydrophobic spliced variant Aβ42^1^. Aβ proteins are released into the extracellular space and can circulate through the bloodstream. Also found in the extracellular space, exosomes are nanoscale membrane vesicles secreted by mammalian cells. During exosome biogenesis, glycoproteins and glycolipids are incorporated into the invaginating plasma membrane and sorted into the newly formed exosomes^14^. Through these surface markers^26^, exosomes can bind with extracellular Aβ proteins (Fig. 1a). Multimodal characterization of vesicles derived from neuronal origin (SH-SY5Y cells) confirmed their exosomal morphology, size distribution, molecular composition, and purity^32^ (Supplementary Fig. 1a–d). Transmission electron microscopy analysis of the vesicles further revealed their ability to bind with Aβ42 proteins (Fig. 1b and Supplementary Fig. 1e).

To evaluate exosome-Aβ association, we developed the APEX platform for amplified, multi-parametric profiling of exosome molecular co-localization. The system employs an in situ enzymatic amplification to rapidly grow insoluble optical deposits over sensor-bound exosomes and measures the associated transmission SPR through a periodic lattice of plasmonic nanostructures (Fig. 1c). To complement the APEX enzymatic deposition (which occurs on the sensor’s top surface), we patterned size-matching plasmonic nanoholes in a coupled, double-layered photonic system for enhanced SPR measurements through backside illumination (away from the enzyme activity, Supplementary Table 1). The resultant enzymatic deposition not only stably changes the refractive index for SPR signal amplification, as demonstrated by the red shift in the transmitted light spectrum (spectral shift Δλ, Fig. 1d), but is also spatially defined for molecular co-localization analysis. Scanning electron micrographs of sensor-bound exosomes, before and after APEX amplification, confirmed the localized growth of optical deposits after enzymatic conversion (Supplementary Fig. 2).

Using the developed APEX platform, we measured the association of Aβ proteins with exosomes, directly from clinical blood samples of AD patients and control subjects, and correlated the measurements to PET imaging of brain plaque deposition (Fig. 1e). For high-throughput, multiplexed clinical analysis, we employed advanced fabrication approach (i.e., deep ultraviolet lithography, Supplementary Fig. 3) to prepare sensor microarrays on 8-inch wafers; each wafer could accommodate more than 40 microarray chips, with >2000 sensing elements (Supplementary Fig. 4a). Figure 1f shows a photograph of the developed APEX microarray chip used in this study for parallel measurements. Scanning electron micrographs of the developed sensor showed highly uniform fabrication (Supplementary Fig. 4b).

### Optimized signal amplification for multiplexed profiling

**Fig. 2:**
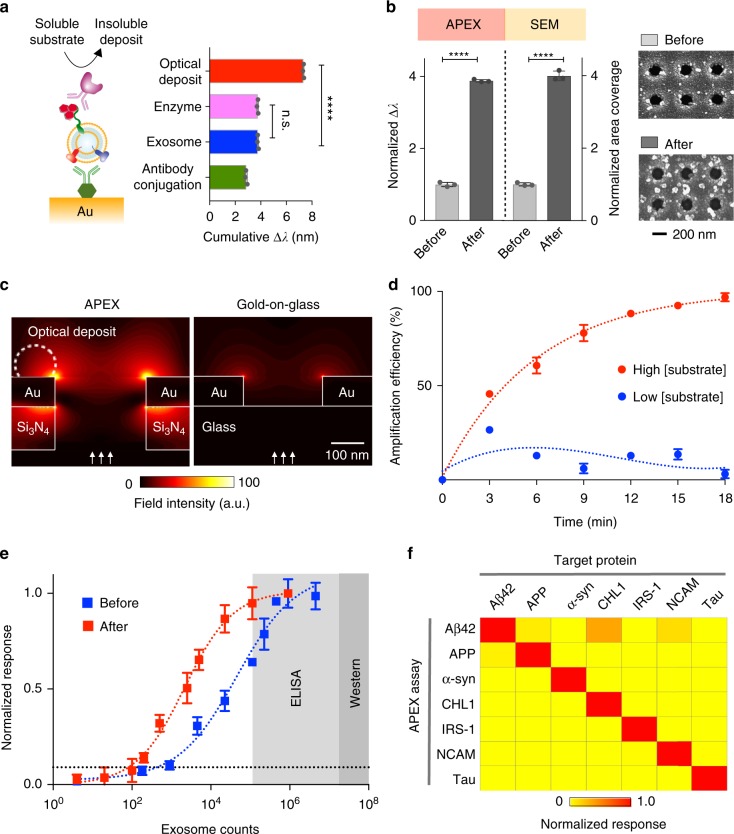
APEX signal amplification and multiplexed profiling. **a** Step-by-step APEX transmission spectral changes. We performed a series of operations, namely antibody conjugation (anti-CD63) onto the sensor, exosome binding, enzyme labeling and enzymatic deposition, and monitored the resultant spectral shifts. While the enzyme labeling did not cause any significant changes, the deposit formation led to ~400% signal enhancement (****P < 0.0001, n.s. not significant, Student’s *t-*test). **b** Comparison of APEX signal amplification and optical deposit area coverage. The increase in area coverage was determined by scanning electron microscopy (SEM) analysis (****P < 0.0001, Student’s *t-*test). All data were normalized to that before the signal amplification. Inserts (right) show SEM images of sensor-bound exosomes, before and after APEX amplification. **c** Finite-difference time-domain simulations with back illumination. The APEX sensor design, but not the gold-on-glass design, enables the generation of enhanced electromagnetic fields through back illumination. Back illumination minimizes direct incident light exposure on the enzyme activity (which occurs on the sensor’s top surface). Arrows indicate the direction of incident illumination. **d** Real-time sensorgrams of APEX amplification kinetics. Different concentrations of the optical substrate (3,3′-diaminobenzidine tetrahydrochloride; high: 1 mg ml^−1^, low: 0.01 mg ml^−1^) were used to monitor the amplification efficiency. All data were normalized against negative controls, performed with IgG isotype control antibodies. **e** Comparison of the detection sensitivities of APEX, ELISA, and western blotting. The APEX detection limit (dotted line), before and after amplification, was determined by titrating a known quantity of exosomes and measuring their CD63 signal. **f** Specificity of APEX assays for measuring target proteins. Assays were developed for amyloid β (Aβ42), amyloid precursor protein (APP), alpha-synuclein (α-syn), close homolog of L1 (CHL1), insulin receptor substrate 1 (IRS-1), neural cell adhesion molecule (NCAM), and tau protein. All assays demonstrated specific detection. Heat map signals were assay (row) normalized. All measurements were performed in triplicate, and the data are displayed as mean ± s.d. in (**a**, **b**, **d**, and **e**). Source data are provided as a Source Data file

We first developed the enzymatic APEX amplification. We performed a series of sensor functionalization, namely antibody conjugation, exosome binding, enzyme labeling, and enzymatic deposition, and measured the step-by-step total spectral shifts (cumulative Δλ, Fig. 2a). We functionalized the sensor with antibodies against CD63, a type III lysosomal membrane protein abundant in and characteristic of exosomes^21,22^, to capture vesicles derived from neuronal cells (SH-SY5Y). To facilitate localized deposition of insoluble optical products, we incorporated horseradish peroxidase as the cascading enzyme and used its soluble substrate (3,3′-diaminobenzidine tetrahydrochloride) to catalyze the conversion. The sensor-bound vesicles were enzyme-labeled via another anti-CD63 antibody. While the enzyme labeling did not cause any significant spectral changes, the enzymatic deposition led to ~400% signal enhancement. In comparison, the control experiment with IgG isotype control antibodies demonstrated minimal background changes (Supplementary Fig. 5). Importantly, this SPR signal amplification correlated well with the increase in area coverage by the localized optical deposits, as confirmed by scanning electron microscopy (Fig. 2b).

To complement the enzymatic amplification (which occurs on the sensor’s top surface), we optimized the APEX sensor design to improve its analytical performance and stability. In comparison to the established gold-on-glass design^31^, which supports only front illumination, the APEX double-layered plasmonic structure enables SPR excitation via back illumination (Fig. 2c and Supplementary Table 1). The new optimized design not only showed strong transmission SPR through back illumination (Supplementary Fig. 6a–c), but also demonstrated analytical stability (Supplementary Fig. 6d), likely due to reduced direct incident illumination (i.e., temperature fluctuation) on the enzymatic activity^33^. We further established the APEX assay by optimizing the enzymatic substrate concentration as well as the reaction duration (Fig. 2d). Under constant back illumination, we monitored the real-time spectral changes associated with different substrate concentrations, and found that substantial signal amplification could be accomplished in <10 min, thus enabling the entire APEX workflow to be completed in <1 h.

With these optimized conditions, we next measured the APEX detection sensitivity for exosome quantification. Neuron-derived vesicles (SH-SY5Y) were quantified with standard nanoparticle tracking analysis. Using anti-CD63 antibodies, we performed a titration experiment (Fig. 2e). We determined that the optimized APEX amplification could boost the detection sensitivity by a 10-fold improvement, establishing a limit of detection (LOD) ~200 exosomes. This observed sensitivity is the best LOD reported thus far for bulk exosome measurements^17^, and fares 10^5^- and 10^3^-fold better than western blotting and chemiluminescence ELISA, respectively^21,31^.

Using the microarray APEX platform, we further developed assays for profiling diverse markers associated with neurodegenerative diseases. On the basis of prior reports^5,34,35^, we established specific assays for the following protein markers: amyloid β (Aβ42), amyloid precursor protein (APP), alpha-synuclein (α-syn), close homolog of L1 (CHL1), insulin receptor substrate 1 (IRS-1), neural cell adhesion molecule (NCAM), and tau protein. These assays not only demonstrated good specificity (Fig. 2f) but also enabled highly sensitive protein detection (Supplementary Fig. 7). Importantly, through further development of the APEX assay workflow (Supplementary Fig. 8a), the platform demonstrated signal amplification capacity for detecting both extra- and intravesicular proteins, as well as exosomal miRNAs (Supplementary Fig. 8b). All detection probes used for assay development can be found in Supplementary Table 2.

### Enhanced binding between Aβ aggregates and exosomes

**Fig. 3:**
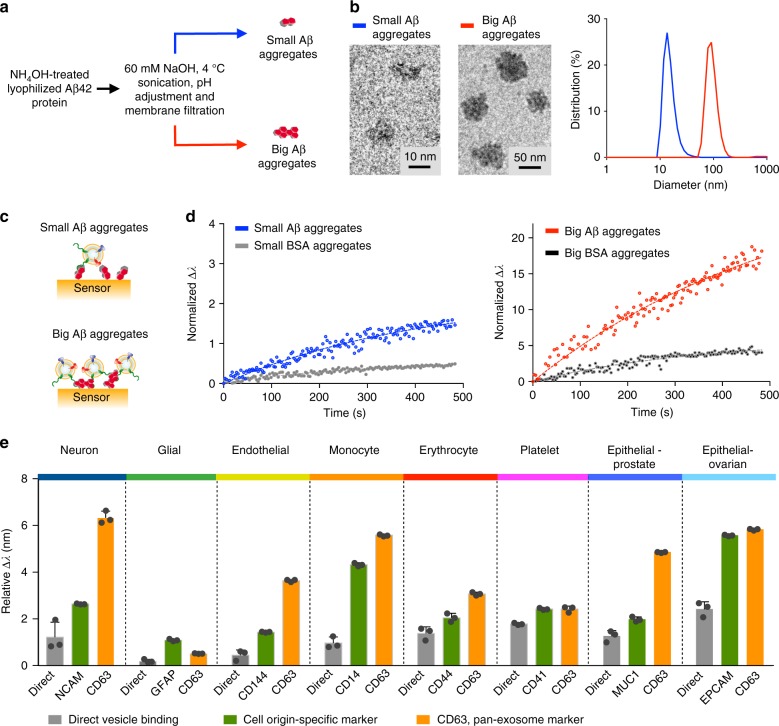
Preferential association between Aβ aggregates and exosomes. **a** Schematics of Aβ protein aggregation. We varied the degree of clustering and used filtration approaches to prepare small and big Aβ42 aggregates, respectively. **b** Characterization of Aβ protein aggregates. (Left) Transmission electron micrographs showed globular morphology of the prepared Aβ42 aggregates. (Right) Dynamic light scattering analysis confirmed the unimodal size distribution of the different-sized preparations. **c** Schematics of the exosome-Aβ association analysis. Aβ42 aggregates (small vs. big) were immobilized onto the APEX sensors and treated with equal concentrations of exosomes derived from neuronal cells (SH-SY5Y) to determine the association kinetics. All exosome binding data were normalized against respective Aβ42 aggregate surface areas immobilized onto the sensors (see Methods for details). **d** Real-time sensorgrams of exosome binding kinetics. In comparison to similar-sized bovine serum albumin (BSA) control aggregates (see Supplementary Fig. 10), exosomes associated more strongly with the Aβ42 aggregates. Importantly, as compared to their binding affinity to the smaller Aβ42 aggregates (left), exosomes demonstrated a much stronger affinity to the bigger Aβ42 aggregates (right). Note the difference in scale on the *y* axis. All binding affinities (*K*_*D*_) were determined from normalized exosome binding data and relative to BSA controls. *K*_D(small)_ / *K*_D(big)_ = 5.27. **e** Differential association of various extracellular vesicles to Aβ42 aggregates. Vesicles were derived from different cell origins, namely neurons, glial cells, endothelial cells, monocytes, erythrocytes, platelets, and epithelial cells, respectively, and used in equal concentrations for the binding analysis. Employing the APEX platform, we first measured the vesicles’ direct binding with the Aβ42-functionalized sensor (direct). Next, for each cell origin, we labeled the bound vesicles for origin-specific marker (cell origin-specific marker) or pan-exosome marker (i.e., CD63, pan-exosome marker), and measured the associated APEX signal amplification. All measurements were made relative to IgG isotype control antibodies, and performed in triplicate. The data are displayed as mean ± s.d. in (**e**). Source data are provided as a Source Data file

Using the developed APEX platform, we next evaluated the association of exosomes to different structural forms of pathological Aβ proteins. To mimic various stages of amyloid seeding and fibrillation^36^, we prepared different-sized aggregates of Aβ42, a major component of amyloid plaques^37,38^. We varied the degree of clustering^39^ to form different-sized Aβ42 aggregates (Fig. 3a, see Methods for experimental details), and confirmed their globular morphology and unimodal size distribution, through transmission electron microscopy and dynamic light scattering analysis, respectively (Fig. 3b). We further noted that the bigger Aβ42 aggregates demonstrated a strong propensity to form fibrillar structures (Supplementary Fig. 9).

To determine the kinetics of exosome-Aβ association, we immobilized the prepared Aβ42 aggregates onto the APEX platform, and incubated the sensors with equal concentrations of neuron-derived exosomes (Fig. 3c). Comparatively, in a control experiment, we prepared and characterized similar-sized aggregates of bovine serum albumin (BSA) (Supplementary Fig. 10). By measuring real-time exosome binding, we demonstrated that regardless of the aggregate size, exosomes could associate more strongly with the Aβ42 aggregates than with similar-sized BSA controls (Fig. 3d). More importantly, in comparison to its binding affinity to the smaller Aβ42 aggregates (Fig. 3d, left), the vesicles showed a significantly higher binding affinity (>5 fold higher) to the bigger Aβ42 aggregates (Fig. 3d, right). All affinities were normalized against Aβ42 aggregate surface areas and made relative to the respective BSA controls (see Methods for details). As the bigger Aβ42 aggregates were also prefibrillar, we reasoned that their enhanced binding to exosomes could be attributed to the aggregates’ differential surface properties and energy states^40,41^.

Next, using extracellular vesicles derived from different cell origins, we employed the APEX platform to measure their respective association with the bigger Aβ42 aggregates (Fig. 3e). Equal concentrations of vesicles derived from various cell origins, as determined by nanoparticle tracking analysis (Supplementary Fig. 11), were incubated with Aβ42-functionalized sensors. We noted that of all the cell origins tested, neuron, erythrocyte, platelet, and epithelial cell-derived vesicles demonstrated stronger association with the Aβ42 aggregates, while glial and endothelial cell-derived vesicles showed negligible binding. We next used a panel of specific markers against these respective cellular origins as well as a pan-exosome marker (i.e., CD63) for APEX signal amplification of the bound vesicles. CD63 performed consistently for signal enhancement across all vesicles tested. With this marker identification, we thus defined exosome-bound Aβ as Aβ42+ CD63+ for subsequent assay development to improve detection coverage (Supplementary Fig. 12a).

### Brain plaque load revealed by blood exosome-bound Aβ measurement

In light of the enhanced binding between exosomes and prefibrillar Aβ aggregates, the building blocks of amyloid plaques, we hypothesized that exosome-bound Aβ could serve as a more reflective circulating biomarker of brain plaque load. To test this hypothesis, we developed various APEX assays with different antibodies to evaluate different populations of circulating Aβ42 from clinical blood samples (Supplementary Fig. 12a). Specifically, to characterize the exosome-bound Aβ42 population, we designed the APEX assay to enrich for Aβ42 directly from native plasma and measure the relative amount of CD63 associated with the captured Aβ42. This assay configuration not only showed specific detection for the Aβ42+ CD63+ population (Supplementary Fig. 12b–c), but also reflected functional relevance: with the enhanced binding between prefibrillar Aβ aggregates and exosomes, the associated exosomal CD63 signal could be considered as a surrogate indicator to measure the relative amount of prefibrillar Aβ42 among total circulating Aβ42. To illustrate the existence of the unbound Aβ42 population, we used size-exclusion filtration to remove large-sized retentate (e.g., exosomes) in plasma before measuring Aβ42 in the plasma filtrate. Finally, to measure total circulating Aβ42, we evaluated native plasma through direct Aβ42 enrichment and Aβ42 detection.

**Fig. 4:**
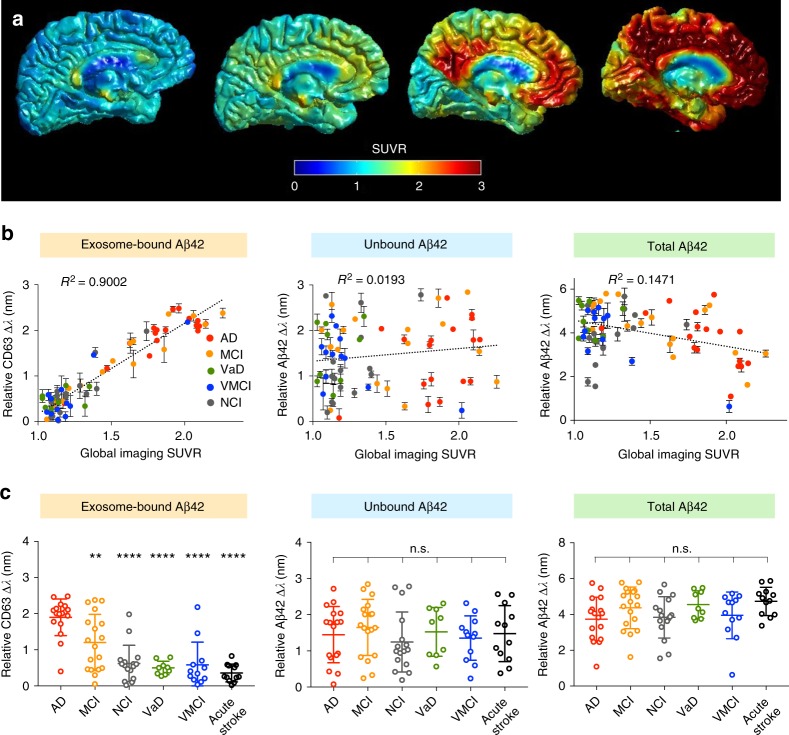
Clinical correlation of circulating exosome-bound Aβ to brain imaging. **a** Representative reconstructed PET brain images from clinical subjects, showing increasing brain amyloid plaque load. SUVR, standardized uptake value ratio, of specific brain region was normalized relative to the mean cerebellar gray matter intensity to determine brain amyloid plaque load. **b** Correlations of different populations of circulating Aβ42 with global average PET brain imaging (*n* = 72). Using the APEX assays, we measured the respective signals from exosome-bound Aβ42 (left), unbound Aβ42 (center), as well as total Aβ42 (right), in blood samples of patients with Alzheimer’s disease (AD), mild cognitive impairment (MCI) as well as control subjects with no cognitive impairment (NCI), vascular dementia (VaD), and vascular mild cognitive impairment (VMCI). When correlated to the global imaging data of brain amyloid plaque, the exosome-bound Aβ42 measurements demonstrated the best correlation (left, *R*^*2*^ = 0.9002), as compared to that of the unbound Aβ42 population (center, *R*^*2*^ = 0.0193) or the total Aβ42 (right, *R*^*2*^ = 0.1471). **c** Analysis of different populations of circulating Aβ42 in distinguishing various clinical groups (*n* = 84). Only APEX measurements of circulating exosome-bound Aβ42 (left) could distinguish between the AD clinical groups (AD and MCI), as well as from other normal (NCI) and clinical controls (VaD, VMCI and acute stroke). Neither the unbound Aβ42 measurements (center) nor the total Aβ42 measurements (right) showed any statistical significance among the different clinical groups (**P < 0.01, ****P < 0.0001, n.s., not significant, Student’s *t*-test). All measurements were made relative to IgG isotype control antibodies, and performed in triplicate. The data are displayed as mean ± s.d. in (**b**, **c**). Source data are provided as a Source Data file.

We next conducted a feasibility clinical study aimed at addressing the following key questions: (1) can APEX measure circulating Aβ42 directly from blood samples, (2) how correlative are different populations of blood-borne Aβ42 with brain plaque load, and (3) can specific populations of circulating Aβ42 distinguish various clinical groups? To achieve these goals, we recruited age-matched subjects (*n* = 84) from multiple independent cohorts and across a wide clinical spectrum. Subjects were diagnosed as AD (*n* = 17), mild cognitive impairment (MCI, *n* = 18), healthy controls with no cognitive impairment (NCI, *n* = 16), as well as clinical controls with vascular dementia (VaD, *n* = 9), and neurovascular compromises (i.e., vascular mild cognitive impairment, VMCI, *n* = 12; and acute stroke, *n* = 12). All clinical information can be found in Supplementary Table 3. All subjects were recruited for blood sampling and APEX analysis. With the exception of the acute stroke patients, all subjects also consented to concurrent PET imaging of brain amyloid plaque. Across and within the imaged clinical groups, PET imaging revealed a broad range of brain plaque load (Fig. 4a), consistent with other published clinical imaging studies^6^.

Using the developed APEX assays (Supplementary Fig. 12a), we evaluated different populations of circulating Aβ42 in these clinical plasma samples, namely the exosome-bound Aβ42, the unbound Aβ42, as well as the total circulating Aβ42 (Fig. 4b). The exosome-bound Aβ42 population showed strong co-localization signals with exosomal markers (i.e., CD63, CD9, and CD81) and neuronal markers (i.e., NCAM, L1CAM, and CHL-1), suggesting that neuronal exosomes could constitute a substantial proportion of the population (Supplementary Fig. 13a). As the unbound Aβ42 measurements were performed from plasma filtrates, we further characterized these filtrates and confirmed their negligible vesicle counts and minimal co-localization signals with exosomal and neuronal markers (Supplementary Fig. 13b). When correlated to global PET amyloid imaging, the exosome-bound Aβ42 measurements showed the best correlation (Fig. 4b, left, *R*^*2*^ = 0.9002), as compared to that of the unbound Aβ42 (Fig. 4b, center, *R*^*2*^ = 0.0193) or the total circulating Aβ42 (Fig. 4b, right, *R*^*2*^ = 0.1471). Interestingly, unlike the poor and negative association demonstrated by the total Aβ42 measurements (as shown in the present study as well as other published reports^12,42^), the relative CD63 measurements from the exosome-bound Aβ42 population showed a correlated and positive association to PET imaging of brain amyloid plaque. We attribute this finding to the similar binding preferences of exosomes and PET tracers to Aβ42: (1) exosomes show enhanced association with prefibillar Aβ42 aggregates, particularly bigger aggregates which can readily form fibrils (Fig. 3d), and (2) PET tracers avidly bind to larger amyloid fibrils, but show little binding to smaller aggregates^43^. Notably, this superior correlation also demonstrated brain region specificity; the exosome-bound Aβ42 measurements showed a stronger correlation to the brain plaque load in the cingulate region (early AD-affected region, Supplementary Fig. 14a) than to that of the occipital region (late AD-affected region, Supplementary Fig. 14b).

On distinguishing clinical diagnoses, only APEX analysis of the exosome-bound Aβ42, but not that of the unbound population or the total plasma Aβ42, demonstrated good specificity (Fig. 4d). In particular, the exosome-bound Aβ42 measurements could differentiate not only between the AD clinical groups (i.e., AD and MCI, *P* < 0.01), but also from other healthy and clinical controls (*P* < 0.0001, Student’s *t*-test). This demonstrated specificity is comparable to that of the PET brain amyloid imaging in distinguishing the various clinical groups (Supplementary Fig. 15). Nanoparticle tracking analysis of the plasma extracellular vesicles, on the other hand, did not reveal any significant difference in vesicle size or concentration across all clinical groups (Supplementary Fig. 16).

## Discussion

AD is the most common form of severe dementia. Due to its complex and progressive neuropathology, early detection and timely intervention are essential to the success of disease-modifying therapies. Despite intense interests in developing blood-based AD measurements, their progress has been confounded by limited sensitivity and poor correlation to brain pathology. Motivated by recent findings that circulating exosomes could bind to pathological AD proteins and mediate disease progression, we hypothesized that exosome-bound Aβ, as a subpopulation of total circulating Aβ proteins, could better reflect brain plaque load.

We thus developed a dedicated analytical platform (APEX) for multi-parametric analysis of exosome-bound Aβ directly from blood plasma (Supplementary Fig. 12a). Designed to overcome the previously mentioned challenges in measuring different populations of circulating Aβ, the APEX is uniquely designed to achieve sensitive nanoscale detection. Specifically, it leverages new advances in assay development, sensor design and device fabrication to enhance detection capabilities (Supplementary Table 1). In terms of assay technology, the APEX platform exploits a rapid, in situ enzymatic conversion to achieve a highly localized, amplified signal. This development not only enables sensitive detection, but also facilitates exosome co-localization analysis for multiplexed population studies, as the insoluble deposits are locally formed only when multiple targets are concurrently found in exosomes. In terms of sensor design, its size-matching, double-layered nanoplasmonics is optimized for the detection of exosome-bound Aβ. The APEX platform comprises a periodic array of gold nanoholes, suspended on a patterned silicon nitride membrane. This double-layered plasmonic structure enables SPR excitation via backside illumination, thereby complementing the APEX assay by reducing direct incident illumination (i.e., temperature fluctuation) on the enzyme activity. In addition, the APEX platform can be mass-fabricated through deep ultraviolet lithography, the state-of-the-art fabrication process for large-scale, precise nano-patterning. Through these integrated advances, the observed APEX sensitivity is thus the best reported so far for bulk exosome profiling and surpasses standard ELISA measurements by several orders of magnitude. Using the developed platform, we demonstrated the enhanced binding between exosomes and bigger prefibrillar Aβ, a key building block of fibrillar amyloid plaque. We further identified and evaluated subpopulations of exosome-bound amyloid (Aβ42+ CD63+) in clinical plasma samples, which we found to be correlative to brain amyloid plaque burden, across a diverse clinical population.

Evaluating different populations of circulating Aβ could lead to new advances in AD research and clinical care. Accumulating evidence supports the potential role of prefibrillar Aβ aggregates as toxic drivers of AD neurodegeneration^37,38^. Their preferential association with exosomes, as shown in the current study, as well as recent findings on the enrichment of exosomal markers in human amyloid plaques^27^, not only sheds light on possible new mechanisms of plaque seeding, but also suggests the importance of exosome-bound Aβ as a more reflective circulating biomarker of the complex AD pathology. We thus envision that the present study could complement other pre-clinical and clinical studies, with respect to both technology development as well as biomarker refinement. In terms of technology development, for example, while mass spectrometry enables unbiased molecular screening and is valuable for biomarker discovery, especially in detecting different molecular isoforms and variants (e.g., (APP)669–711 and Aβ1–40)^11,12^, the APEX technology provides rapid and sensitive readouts directly from native plasma samples, without requiring extensive sample processing that is typically necessary for mass spectrometry measurements, and is thus suitable for direct clinical measurements. In terms of biomarker refinement, as demonstrated by the current study, analysis of different populations of circulating Aβ (e.g., molecular and organizational) could reveal novel correlations previously masked by ensemble blood measurements, and advance future blood-based clinical management of AD.

With more than 400 AD clinical trials at present^44^, we also envision that the current platform could be further developed to improve the standard-of-care for patients. While the current study uses CD63 for exosome identification, combinatorial analysis of additional markers (e.g., neuronal markers, AD markers^45,46^) could identify new biomarker subpopulations and develop valuable composite signatures. Through additional technical innovations, such as on-chip exosome processing^17^, the technology could be further expanded to accelerate large cohort clinical validations. These advances will provide new opportunities to facilitate minimally-invasive detection and molecular stratification, all of which are important for objective evaluation of disease-modifying therapies at different stages of clinical trials.

## Methods

### Cell culture

Human cell lines SH-SY5Y (neuron), HUVEC (umbilical vein endothelial), THP-1 (monocyte), PC-3 (prostate epithelial), and SK-OV-3 (ovarian epithelial) were obtained from American Type Culture Collection. GLI36 (glia) and SK-OV-3 were grown in Dulbecco’s Modified Essential Medium (DMEM, Gibco). SH-SY5Y was cultured in Dulbecco’s Modified Eagle Medium: Nutrient Mixture F-12 Medium (DMEM/F12 Gibco). PC-3, THP-1 and HUVEC were grown in F-12K, RPMI-1640 and EGM-2 media, respectively. With the exception of EGM-2, which was supplemented with 5% fetal bovine serum (FBS), all other media were supplemented with 10% FBS and penicillin-streptomycin.

### Exosome isolation and quantification

Cells at passages 1–15 were cultured in vesicle-depleted medium (with 5% depleted FBS) for 48 h before vesicle collection. All media containing exosomes were filtered through a 0.2-μm membrane filter (regenerated cellulose, Millipore), isolated by differential centrifugation (first at 10,000 × *g* and subsequently at 100,000 × *g*)^21^, and used for exosome analysis with the APEX platform. For exosome isolation from blood cells and platelets, blood cells were derived from blood fractionation and platelets from platelet-rich plasma. All vesicles were collected as previously described^47^. For independent quantification of exosome concentration, we used the nanoparticle tracking analysis (NTA) system (NS300, Nanosight). Exosome concentrations were adjusted to obtain ~50 vesicles in the field of view to achieve optimal counting. All NTA measurements were done with identical system settings for consistency. Note that exosome isolation was only performed for cell culture experiments. Clinical measurements were performed on native plasma samples, without needing for any vesicle isolation.

### APEX sensor fabrication

APEX sensors were fabricated on 8-inch silicon (Si) wafers. Briefly, a 10-nm silicon dioxide (SiO_2_) layer was prepared through thermal oxidation and a 145-nm silicon nitride (Si_3_N_4_) was deposited on the wafer through low pressure chemical vapor deposition (LPCVD). After coating with photoresist, deep ultraviolet (DUV) lithography was performed to define the nanohole array pattern in the resist. This pattern was transferred via reactive ion etching (RIE) to the Si_3_N_4_ membrane. After removing the photoresist, a thin protective layer (100 nm) of SiO_2_ was deposited on the front side of the wafer using plasma enhanced chemical vapor deposition (PECVD). To enable light transmission, the backside of the wafer was spin-coated with photoresist; lithography method was used to define the sensing area. Si_3_N_4_ and SiO_2_ were etched by RIE and followed by potassium hydroxide (KOH) and tetramethylammonium hydroxide (TMAH) etching of Si. After etching, diluted hydrogen fluoride (DHF) (1:100) was used to remove the protective SiO_2_ layer. Finally, Ti/Au (10 nm/100 nm) were deposited onto the Si_3_N_4_ membrane. All nanohole dimension and sensor uniformity were characterized by scanning electron microscopy (JEOL 6701).

### Channel assembly

Standard soft lithography was used for fabricating a multi-channel flow cell. We used SU-8 negative resist (SU8-2025, Microchem) to prepare the mold. The photoresist was spin-coated onto a Si wafer at 2000 rpm for 30 s, and baked at 65 °C and 95 °C for 2 min and 5 min, respectively. After UV light exposure, the resist was baked again before being developed under agitation. The developed mold was chemically treated with trichlorosilane vapor inside a desiccator for 15 min before subsequent use. Polydimethylsiloxane polymer (PDMS) and cross-linker were mixed at a ratio of 10:1 and casted onto the SU-8 mold. After curing at 65 °C for 4 h, the PDMS layer was cut from the mold and assembled onto the APEX sensor. All inlets and outlets were made with 1.1-mm biopsy punch for sample processing.

### Optical analysis

Full 3D finite-difference time-domain (FDTD) simulations were performed using a commercial software package (FDTD solutions, Lumerical). APEX sensors were illuminated with a plane wave from the bottom side (away from enzyme activity). A uniform mesh of 2 nm was applied in all directions. For experimental analysis, a tungsten halogen lamp (StockerYale Inc.) was used to back illuminate the APEX sensor through a ×10 microscope objective. Transmitted light was collected by an optical fiber and fed into a spectrometer (Ocean Optics). All measurements were performed at room temperature, in an enclosed box to eliminate ambient light interference. The transmitted light intensity was digitally recorded in counts against wavelength. For spectral analysis, the spectral peaks were determined using a custom-built R program by fitting the transmission peak using local regression method. All fittings were done locally. That is, for the fit at point x, the fit is made using points near x, weighted by their distance from x. In comparison to fitting by multi-order polynomial curve, this method could eliminate the result variation caused by the number of data points and data range being analyzed. In determining the optimal sensor geometry (Supplementary Fig. 6), we used the spectral changes to quantify peak transmission intensity, peak shape (full width at half maximum, FWHM), and detection sensitivity in response to refractive index changes, respectively. The measured transmission spectra demonstrated uniformity across different sensors, with a s.d. of 0.03 nm in baseline spectral peak positions. All spectral shifts (Δλ) were determined as changes in the transmission spectral peaks, and calculated relative to appropriate control experiments (see below for details).

### Sensor surface functionalization

To confer molecular specificity on the APEX sensor, the fabricated Au surface was first incubated with a mixture of polyethylene glycol (PEG) containing long active (carboxylated) thiol-PEG and short inactive methylated thiol-PEG (Thermo Scientific) (1:3 active: inactive, 10 mM in PBS) for 2 h at room temperature. After washing, the surface was activated through carbodimide crosslinking, in a mixture of excess NHS/EDC dissolved in MES buffer, and conjugated with specific probes and ligands (e.g., antibodies and Aβ42 aggregates). All probe information can be found in Supplementary Table 2. Excess unbound probes were removed by PBS washing. Conjugated sensors were stored in PBS at 4 °C for subsequent use. All sensor surface modifications were spectrally monitored to ensure uniform functionalization.

### APEX signal amplification

To establish the APEX amplification, we incorporated enzymatic growth of insoluble optical deposits for signal enhancement, and optimized the substrate concentration and reaction duration to establish the platform. Briefly, exosomes were incubated for 10 min with the CD63-functionalized APEX sensor (BD Biosciences). The bound vesicles were then labeled with biotinylated anti-CD63 antibody (Ancell, 10 min). As a control experiment, an equivalent amount of biotinylated IgG isotype control antibody (Biolegend) was used on the bound vesicles to determine the amplification efficiency. After washing of unbound antibodies, high sensitivity horseradish peroxidase, conjugated with neutravidin (Thermo Scientific), was allowed to react with the bound vesicles, before the introduction of different concentrations of 3,3′-diaminobenzidine tetrahydrochloride (Life Technologies) as the reaction substrate. Real-time spectral changes were monitored to determine the optimal substrate concentration and reaction duration. The optimized conditions were determined to be 1 mg ml^−1^ for 3 min. All flow rates for incubation and washing were maintained at 3 μl min^−1^ and 10 μl min^−1^, respectively. Localized deposition of insoluble enzymatic products was confirmed through scanning electron microscopy. This optimized workflow is illustrated in Supplementary Fig. 8a.

### Enzyme-linked immunosorbent assay (ELISA)

Capture antibodies (5 μg ml^−1^) were adsorbed onto ELISA plates (Thermo Scientific) and blocked with Superblock (Thermo Scientific) before incubation with samples. After washing with PBST (PBS with 0.05% Tween 20), detection antibodies (2 μg ml^−1^) were added and incubated for 2 h at room temperature. Following incubation with horseradish peroxidase-conjugated secondary antibody (Thermo Scientific) and chemiluminescent substrate (Thermo Scientific), chemiluminescence intensity was measured for protein detection (Tecan).

### Western blotting

Exosomes isolated by ultracentrifugation were lysed in radio-immunoprecipitation assay (RIPA) buffer containing protease inhibitors (Thermo Scientific) and quantified using bicinchoninic acid assay (BCA assay, Thermo Scientific). Protein lysates were resolved by sodium dodecyl sulfate polyacrylamide gel electrophoresis (SDS-PAGE), transferred onto polyvinylidene fluoride membrane (PVDF, Invitrogen), and immunoblotted with antibodies against protein markers: HSP90 (Cell Signaling), HSP70 (BioLegend), Flotillin 1 (BD Biosciences), CD63 (Santa Cruz), ALIX (Cell Signaling), TSG101 (BD Biosciences), LAMP-1 (R&D Systems), and neuronal marker NCAM (R&D Systems), APOE (Abcam), Calnexin (Invitrogen), GRP94 (Santa Cruz). Following incubation with horseradish peroxidase-conjugated secondary antibody (Cell Signaling), enhanced chemiluminescence was used for immunodetection (Thermo Scientific).

### Protein aggregation

Lyophilized NH_4_OH-treated Aβ42 protein (rPeptide) was resuspended in NaOH (60 mM, 4 °C), sonicated, and pH adjusted to pH 7.4 in PBS^39^. The protein was immediately filtered through a 0.2-μm membrane filter (Millipore) and the filtrate was used as the smaller Aβ42 aggregates. For preparation of the bigger Aβ42 aggregates, the protein was treated as described above and incubated with agitation for 1 h to induce further aggregation, before being filtered through a 0.2-μm membrane filter (Millipore). The filtrate was used as the big Aβ aggregates. To prepare similar-sized BSA aggregates as controls, 2% w/v BSA was dissolved in PBS, heated at 80 °C for 1 h and 2 h to induce clustering of the small and big control aggregates, respectively.

### Aggregate size determination

Hydrodynamic diameter of Aβ42 and BSA aggregates were determined by dynamic light scattering analysis (Zetasizer Nano ZSP, Malvern). 3 × 14 measurement runs were performed at 4 °C. Z-average diameter and polydispersity were analyzed. For every measurement, the autocorrelation function and polydispersity index were monitored to ensure sample quality for size determination.

### Characterization of exosome-Aβ association

The prepared protein aggregates (Aβ42 and BSA control) were used for surface functionalization onto the APEX sensors, via EDC/NHS coupling as previously described. Unbound protein aggregates were washed away with PBS. The amount of protein conjugated was measured from the resultant transmission spectral shifts. We used this information to determine the number of conjugated protein aggregates and their associated total protein surface area for exosome binding (see below for detailed information), so as to normalize for binding affinities. Following surface functionalization with the protein aggregates, exosomes were introduced onto the sensors. Spectral changes were measured every 3 s for a total duration of 480 s to construct real-time kinetics sensorgrams. Exosome association kinetics and binding affinities were determined for different-sized protein aggregates.

To account for protein aggregate size differences, as well as SPR-associated exponential decay of sensitivity (with increasing distance from the sensor surface), the following equation was used to calculate the total surface area of the conjugated aggregates for interaction with exosomes:

$$S = E \times {\int}_0^{2r} {\pi \left[ {r^2 - \left( {r - z} \right)^2} \right] \times e^{\left( { - 2z/l_d} \right)}dz}$$

where *S* is the signal, *z* is the distance from the sensor surface, *E* is the electric field at *z* = 0 and a constant in this case, *l*_d_ is the decay length and is set at 200 nm with the current sensor design, *r* is the radius of the conjugated protein aggregate.

All protein aggregates were approximated as spheres, as supported by transmission electron micrographs (Fig. 3b, left) and their *r* determined from dynamic light scattering analysis. We used the above equation to determine the number of protein aggregates conjugated onto the sensor, as well as their respective total surface area, to estimate the number of available binding sites for interaction with exosomes. All exosome binding data (Δλ) were normalized against their respective protein binding sites. Normalized Aβ42 binding data were made relative to similar-sized BSA controls and fitted to determine the binding affinity constant *K*_*D*._

### Scanning electron microscopy

All samples were fixed with half-strength Karnovsky’s fixative and washed twice with PBS. After dehydration in a series of increasing ethanol concentrations, samples were transferred for critical drying (Leica) and subsequently sputter-coated with gold (Leica), before imaging with a scanning electron microscope (JEOL 6701).

### Transmission electron microscopy

Gold nanoparticles (Ted Pella) were used for immuno-labeling of exosomes (CD63, 20 nm) and Aβ42 protein (10 nm or 5 nm in double immuno-labeling). All samples were fixed with 2% paraformaldehyde and transferred onto a copper grid (Ted Pella). The bound samples were washed and contrast-stained with uranyl oxalate and methyl cellulose mixture. Dried samples were imaged with a transmission electron microscope (JEOL 2200FS).

### Clinical sample collection

The study was approved by NUH Institutional Review Board (2015/00441, 2015/00406 and 2016/01201). Subjects were recruited from multiple independent cohorts, according to Institutional Review Board approved protocols. All recruited subjects underwent cognitive assessments at the National University Hospital (NUH, Singapore), including Mini Mental State Examination (MMSE), Montreal Cognitive Assessment (MoCa), and Vascular Dementia battery (VDB). Clinical diagnosis of AD, MCI, or NCI was made after a standardized clinical evaluation. Clinical diagnosis of VaD and VMCI was made from a combination of cognitive assessments, clinical history of stroke, and degree of cerebrovascular disease observed through Magnetic Resonance Imaging (MRI). All clinical assessments and classification were performed according to published criteria^48–50^. Acute stroke plasma samples were collected from patients within 24 hours of hospital admission with a diagnosis of stroke. For plasma collection, venous blood (5 ml) was drawn from subjects, prior to infusion of PET radiotracer (where applicable), in EDTA tubes and processed immediately. Briefly, all blood samples were centrifuged for 10 min at 400 × *g* (4 °C). Plasma was transferred without disturbing the buffy coat and centrifuged again for 10 min at 1100 × *g* (4 °C). All plasma samples were de-identified and stored at −80 °C before APEX measurements. All APEX measurements were performed blinded from PET imaging results and clinical diagnoses.

### Clinical APEX measurements

All plasma samples were measured according to the assay configurations outlined in Supplementary Fig. 12a. Briefly, to measure the exosome-bound Aβ42 population, we used native plasma samples directly, through Aβ42 capture and CD63 detection (Aβ42+ CD63+), without requiring any vesicle purification or isolation. To illustrate the existence of the unbound Aβ42 population in plasma samples, we used size-exclusion filtration (cutoff size = 50 nm, Whatman) to remove large-sized retentate. This was necessary as the assay configuration, based on Aβ42 capture and Aβ42 detection, could not differentiate between the unbound from the total Aβ42. To measure the unbound Aβ, we evaluated the plasma filtrate through Aβ42 capture and Aβ42 detection (Aβ42+ Aβ42+). Note that this filtration was only performed to demonstrate the presence of the unbound Aβ42 population; it is not necessary in the clinical context where only the more reflective exosome-bound Aβ42 will be measured directly from native plasma samples. To measure total Aβ42, we evaluated native plasma samples directly through Aβ42 capture and Aβ42 detection (Aβ42+ Aβ42+). For all measurements, we used 5% BSA as a blocking agent for the APEX sensor. We also included a sample-matched negative control, where we incubated the same sample over a control sensor functionalized with IgG isotype control antibody. All measurements were made relative to this IgG control to account for sample-matched non-specific binding.

### Positron emission tomography (PET) imaging

Following blood draw, subjects were scanned using a Siemens 3 T Biograph mMR system (Siemens Healthineers) for simultaneous acquisition of PET and MR images. PET data were acquired 40–70 min after an intravenous infusion of 370 MBq of 11C-Pittsburgh Compound B (PiB). MR data were acquired using a 12-channel head receive coil for acquisition and consisted of an Ultrashort Echo Time (UTE) image for PET attenuation correction and a T1-weighted Magnetization Prepared Gradient Echo (MPRAGE) image (1 mm isotropic resolution, TI/TE/TR = 900/3.05/1950 ms).

### PET data analysis

T1-weighted MPRAGE images were processed using Freesurfer (5.3.0) to produce parcellations of the cortex for PET data analysis. PET images were reconstructed using an Ordinary Poisson Ordered Subset Expectation Maximization (OP-OSEM) algorithm and smoothed using a 4 mm Gaussian filter. Data were attenuated using the UTE based μ-map. Resulting attenuation corrected Standardized Uptake Value (SUV) images were then co-registered to the MPRAGE images using Advanced Normalization Tools (ANTs) and the subject specific Freesurfer parcellation was used to calculate the Standardized Uptake Value Ratio (SUVR) relative to the mean cerebellar gray matter intensity. Mean SUVRs were calculated for specific regions and the global average SUVR for each patient was calculated by averaging the SUVRs of all brain regions.

### Statistical analysis

All measurements were performed in triplicate and the data are displayed as mean ± s.d. Significance tests were performed via a two-tailed Student’s *t*-test. For inter-sample comparisons, multiple pairs of samples were each tested, and the resulting *P* values were adjusted for multiple hypothesis testing using Bonferroni correction. Values that had an adjusted *P* < 0.05 were determined as significant. For the clinical study, correlations were performed with linear regression to determine the goodness of fit (*R*^*2*^). All statistical analyses were performed using the R-package (version 3.4.2) and Graphpad Prism 7.

## Supplementary information

Supplementary Information

Reporting Summary Checklist

Source Data

## Data Availability

Data supporting the findings of this study are available from the corresponding author on reasonable request. The source data underlying Fig. 2a, b, d, e; 3e and 4b and Supplementary Figs 6a, 8b, 12b, c, 13a, c and 14a, b are provided as a Source Data file.
