# Supplementary material for: Subtyping of circulating exosome-bound amyloid β reflects brain plaque deposition
Source: Nat Commun. 2019 Mar 8;10:1144. doi: 10.1038/s41467-019-09030-2 (PMC6408581; doi:10.1038/s41467-019-09030-2)
Supplement: Supplementary file 1 — Supplementary Information [file 41467_2019_9030_MOESM1_ESM.pdf]

SUPPLEMENTARY INFORMATION

Subtyping of circulating exosome-bound amyloid β reflects brain plaque deposition

Carine Z.J. Lim *et al*.

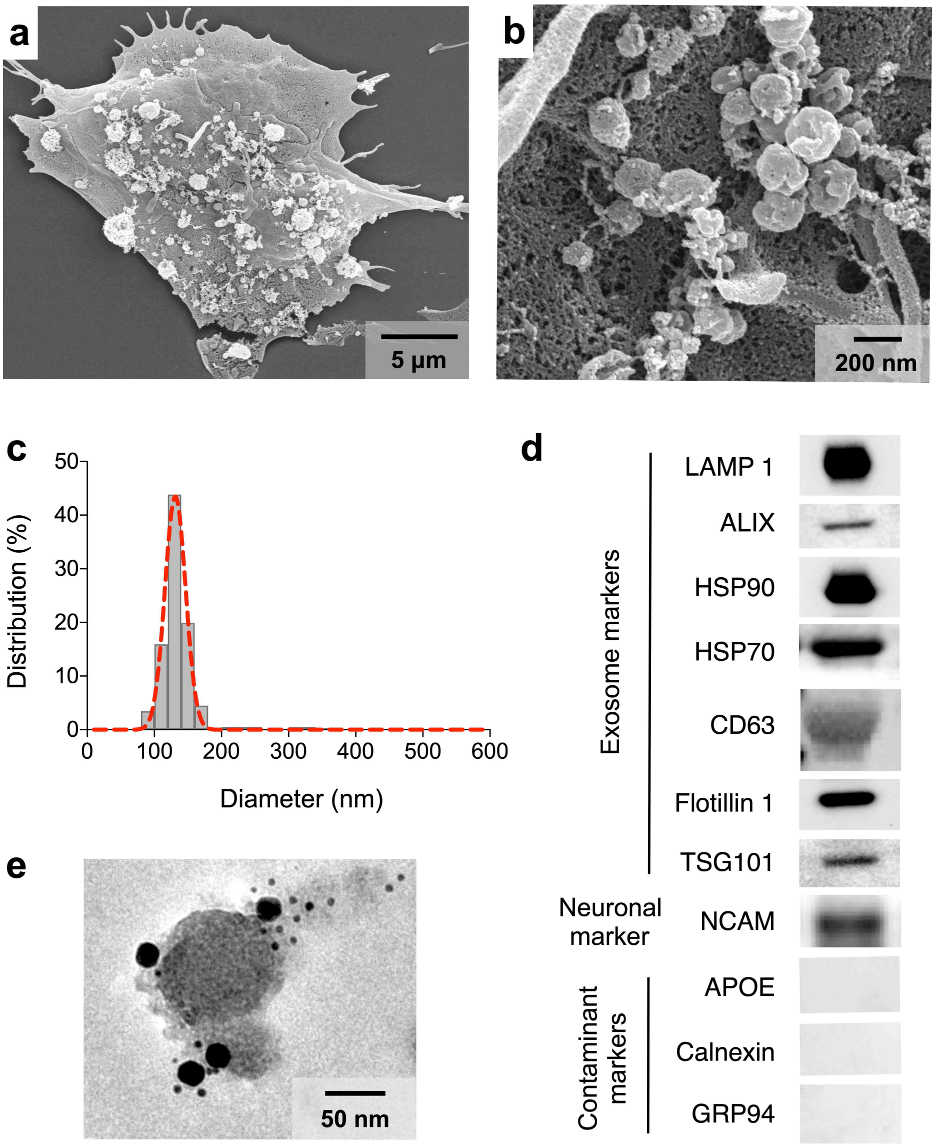

Supplementary Figure 1. Characterization of extracellular vesicles shed by neuronal cells. **(a)** Scanning electron micrograph of a neuronal cell (SH-SY5Y), showing avid release of nanoscale extracellular vesicles from the cell. **(b)** High magnification image of the released vesicles. **(c)** Unimodal size distribution of extracellular vesicles, as determined by nanoparticle tracking analysis, showing a mean diameter of ~ 150 nm. **(d)** Western blotting analysis of the vesicle lysate. The vesicles were lysed and immunoblotted for exosomal markers (LAMP 1, ALIX, HSP90, HSP70, CD63, Flotillin 1, TSG101), neuronal marker (NCAM) as well as negative markers including lipoprotein (APOE) and markers of other membranous compartments (Calnexin, GRP94). **(e)** Transmission electron micrograph of double immuno-labeling with different-sized gold nanoparticles (CD63, 20 nm; Aβ42, 5 nm) confirmed the co-localization of both markers on the same vesicle, illustrating the presence of exosome-bound Aβ.

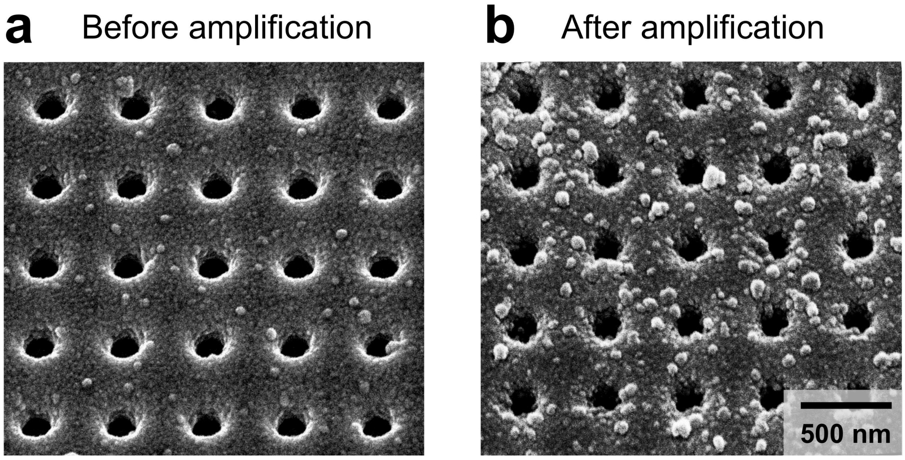

Supplementary Figure 2. APEX amplification product. Scanning electron micrographs of the APEX sensor **(a)** before amplification, with exosomes captured onto the sensor via anti-CD63 antibody, and **(b)** after amplification, showing localized growth of insoluble optical deposits from the soluble substrate (3,3'-diaminobenzidine tetrahydrochloride). The resultant APEX signal amplification correlated well to the increase in area coverage by the localized deposits.

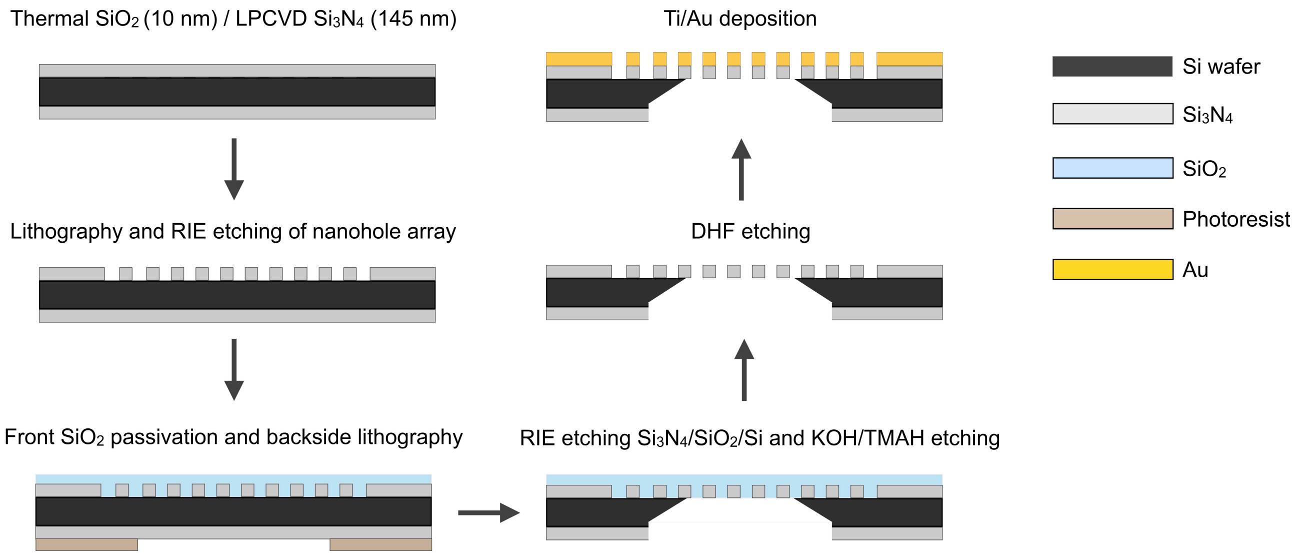

Supplementary Figure 3. Mass fabrication of APEX microarray sensors. All APEX sensors were fabricated on 8-inch silicon (Si) wafer. The fabrication steps include the following: (1) A 10-nm silicon dioxide (SiO_2) layer was prepared through thermal oxidation and a 145-nm silicon nitride (Si_3N_4) was deposited on the wafer through low pressure chemical vapor deposition (LPCVD). (2) After coating with photoresist, deep ultraviolet (DUV) lithography was performed to define the nanohole array pattern in the resist. This pattern was transferred via reaction ion etching (RIE) to the Si_3N_4 membrane. (3) After removing the photoresist, a thin protective layer (100 nm) of SiO_2 was deposited on the frontside of the wafer using plasma enhanced chemical vapor deposition (PECVD). To enable light transmission, the backside of the wafer was spin-coated with photoresist; lithography method was used to define the sensing area. (4) Si_3N_4 and SiO_2 were etched by RIE followed by potassium hydroxide (KOH) and tetramethylammonium hydroxide (TMAH) etching of Si. (5) After etching, diluted hydrogen fluoride (DHF) (1:100) was used to remove the protective SiO_2 layer. (6) Ti/Au (10 nm/100 nm) were deposited onto the Si_3N_4 membrane.

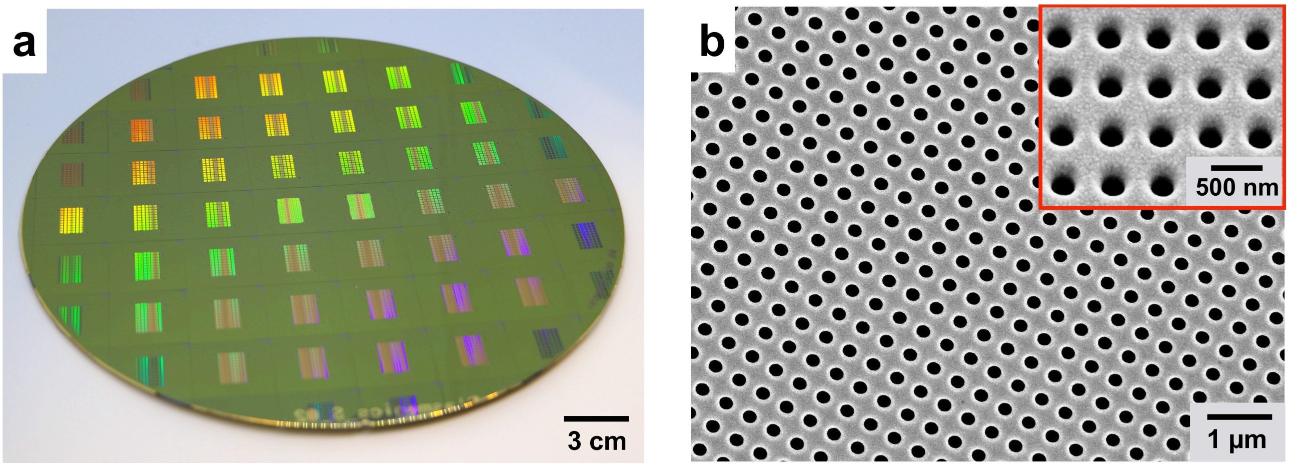

Supplementary Figure 4. Characterization of APEX microarray sensors. (a) Photograph of an 8-inch wafer showing large-scale fabrication of APEX microarray sensor chips. Each wafer consists of >2000 sensing elements. **(b)** Scanning electron micrograph of highly uniform nanoholes in the APEX sensor. Insert shows a magnified view of the nanohole lattice.

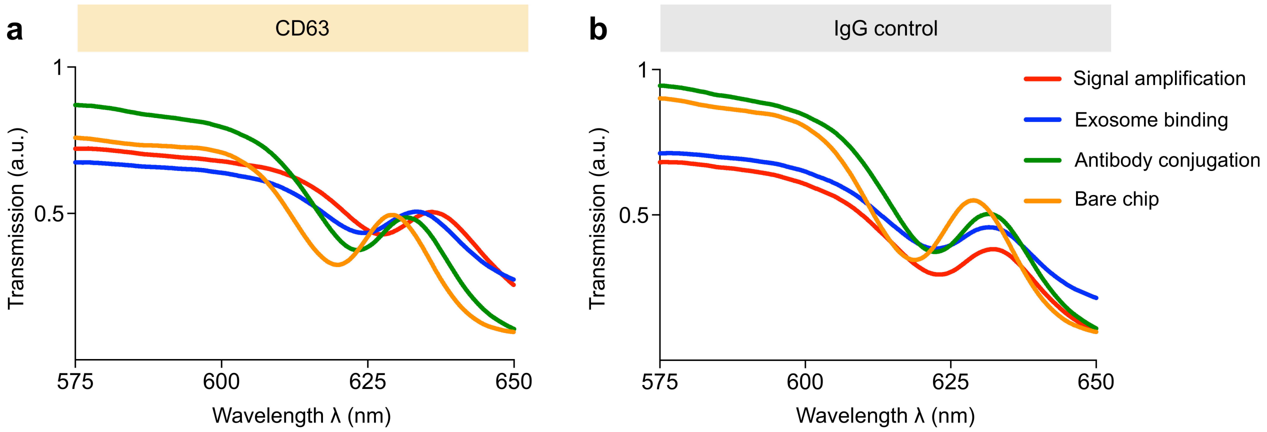

Supplementary Figure 5. Step-by-step spectral changes. APEX sensors were conjugated with either **(a)** anti-CD63 antibody for exosome capture or **(b)** isotype control antibody. All sensors were treated with equal concentrations of exosomes derived from neuronal cell line (SH-SY5Y) before APEX amplification. While the sensors showed a similar degree of surface functionalization with the antibodies (antibody conjugation), only the anti-CD63 functionalized sensor demonstrated significant spectral shifts associated with exosome binding and APEX amplification, respectively. Note that in the control sensor, in the absence of exosome binding, APEX amplification induced negligible spectral change. a.u., arbitrary unit.

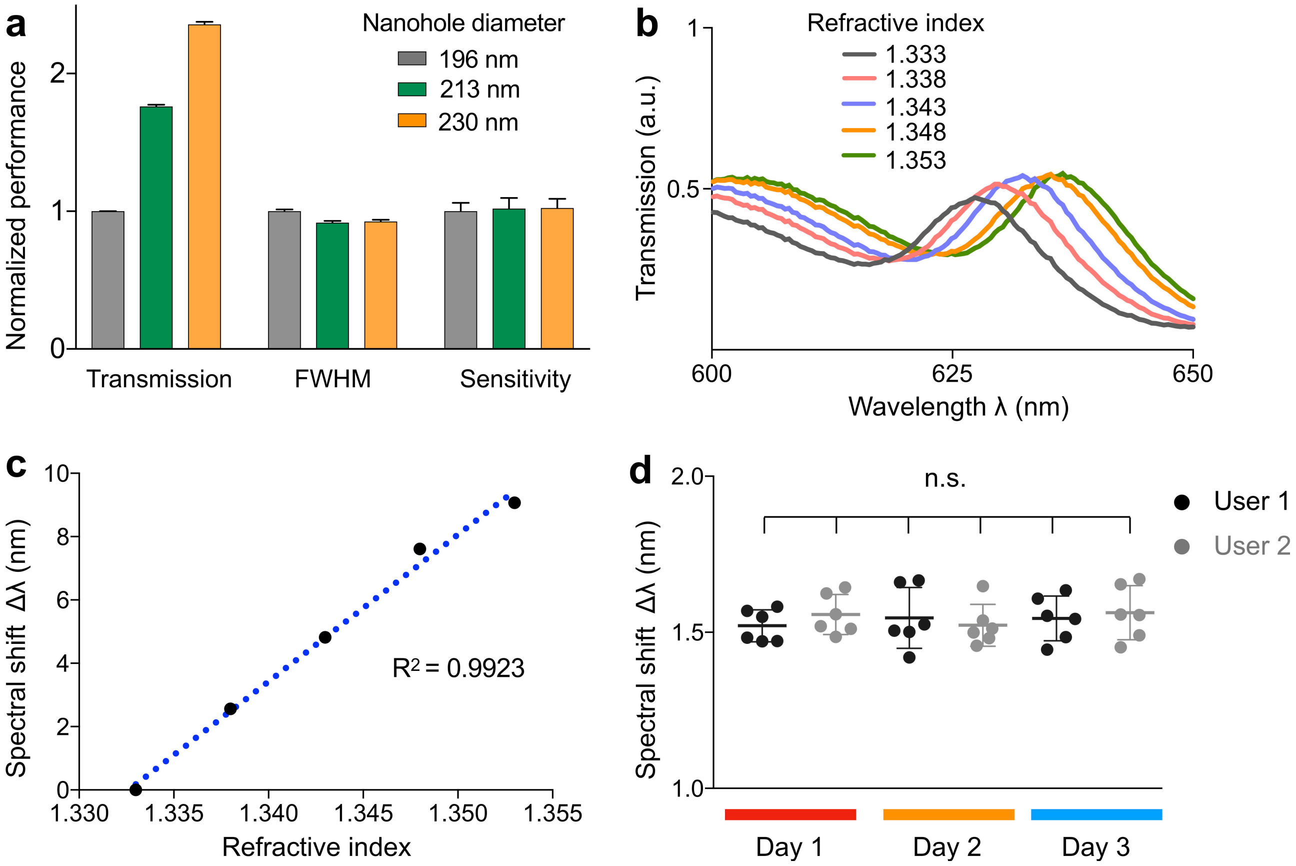

Supplementary Figure 6. Optimization of APEX sensor performance. (a) Comparison of sensor performance with back illumination. We compared the SPR transmission intensity, full width at half maximum (FWHM) of the spectral peak, and detection sensitivity of different APEX sensors with varying nanohole diameter. All sensors were illuminated from the backside to complement the APEX enzymatic amplification. The optimized APEX design has a nanohole diameter of 230 nm, patterned with a regular periodicity of 450 nm in a 100 nm-thick gold layer suspended on a silicon nitride membrane. This double-layered plasmonic structure supports SPR excitation through back illumination. **(b)** Transmission spectral changes of the optimized sensor against increasing refractive index. Increase in refractive index induced a change in the transmission spectrum and shifted the resonance peak to a longer wavelength. **(c)** Spectral shifts showed a linear correlation to increasing refractive index. **(d)** APEX reproducibility and repeatability. APEX enzymatic amplification was performed on the same sample, and measured across different users, sensor chips and time of measurement. The measurements showed the following analytical coefficients of variation: between group = 2.76%, within group = 4.14%, total = 4.59%. All measurements were performed in triplicate or more, and the data are displayed as mean ± s.d. in (a). a.u., arbitrary unit, n.s., not significant, Student's *t*-test.

Target protein

APEX assay	Aβ42	APP	α-syn	CHL1	IRS-1	NCAM	Tau
Aβ42	0.165	N.D.	N.D.	$1x10^6$	N.D.	$1x10^6$	N.D.
APP	N.D.	0.179	N.D.	N.D.	N.D.	N.D.	N.D.
α-syn	N.D.	N.D.	0.133	N.D.	N.D.	N.D.	N.D.
CHL1	N.D.	N.D.	N.D.	0.475	N.D.	N.D.	N.D.
IRS-1	N.D.	N.D.	N.D.	N.D.	0.179	N.D.	N.D.
NCAM	N.D.	N.D.	N.D.	N.D.	N.D.	0.538	N.D.
Tau	N.D.	N.D.	N.D.	N.D.	N.D.	N.D.	0.374

Supplementary Figure 7. Sensitivity of APEX assays. Lowest detectable concentrations of various APEX assays. All detectable concentrations are listed in pg/ml. N.D., non-detectable signal at a sample concentration of >1 ug/ml.

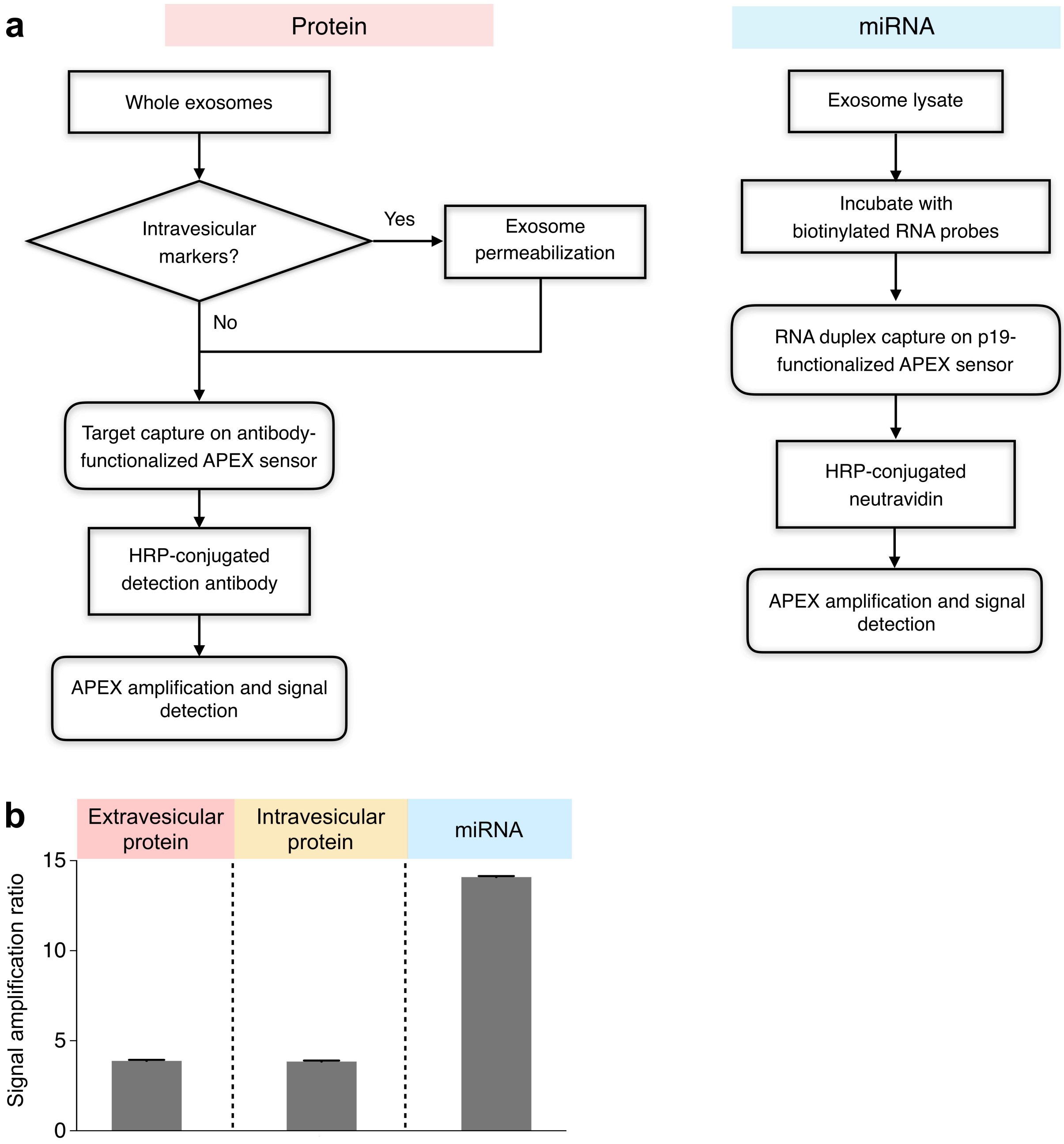

Supplementary Figure 8. APEX workflow and amplification efficiency. (a) APEX workflow for detection of proteins (extra- and intra-vesicular) and miRNA. **(b)** APEX amplification efficiency for different molecular targets. APEX signals were acquired for the following targets: extravesicular protein, Aβ42 protein; intravesicular protein, heat shock protein 90; miRNA, miRNA-9. All signals were normalized to that before the addition of the optical substrate to determine the amplification ratio. Measurements were performed in triplicate, and the data are displayed as mean ± s.d. in (b).

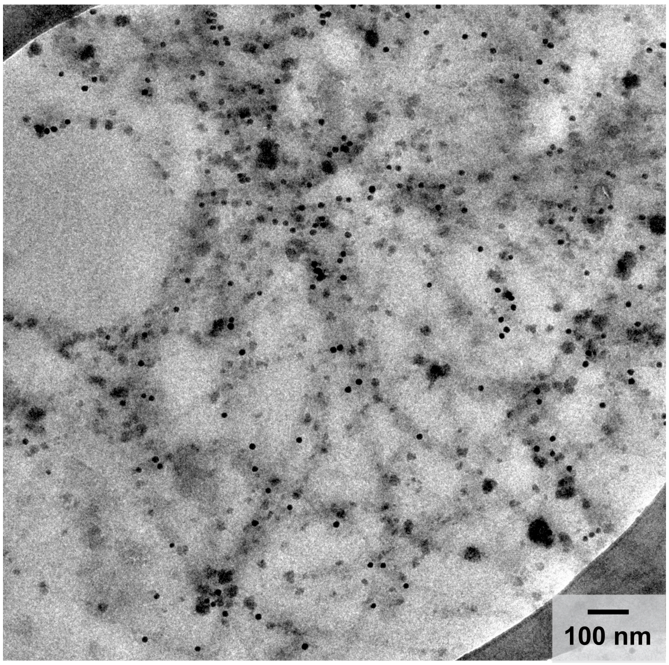

Supplementary Figure 9. Fibrillar structures assembled from big Aβ aggregates. Amyloid fibrils were observed after a 2-hour incubation of the prepared big Aβ42 aggregates. The formed structures were immuno-labeled with gold nanoparticles (10 nm) via anti-Aβ42 antibody and characterized with transmission electron microscopy.

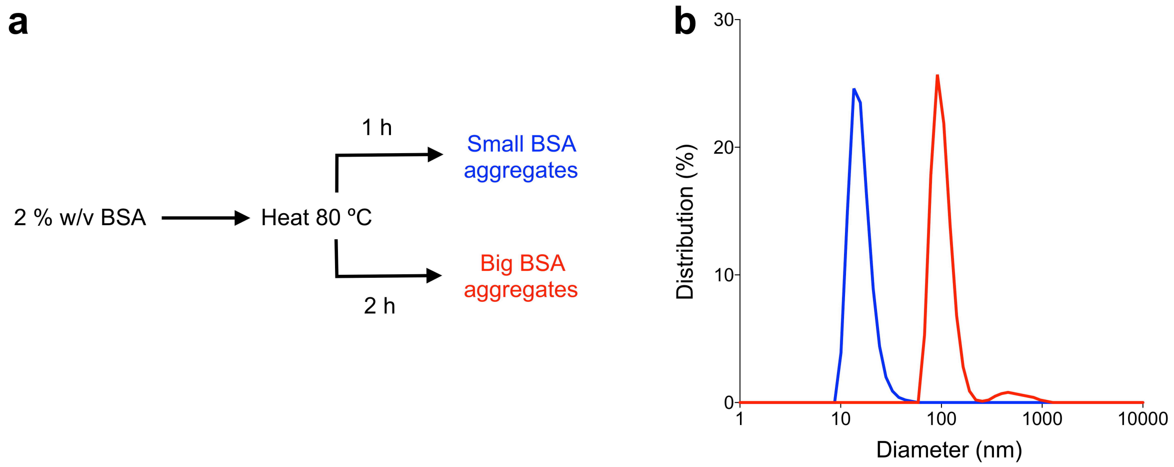

Supplementary Figure 10. Preparation of BSA control aggregates. (a) Schematics of BSA protein aggregation. We varied the duration of heating to prepare small and big BSA control aggregates, respectively. **(b)** Characterization of BSA protein aggregates. The hydrodynamic diameters of the BSA aggregates were determined by dynamic light scattering analysis. Both aggregates showed unimodal size distribution. The small aggregates have a diameter ~ 15 nm and the big aggregates have a diameter ~ 100 nm.

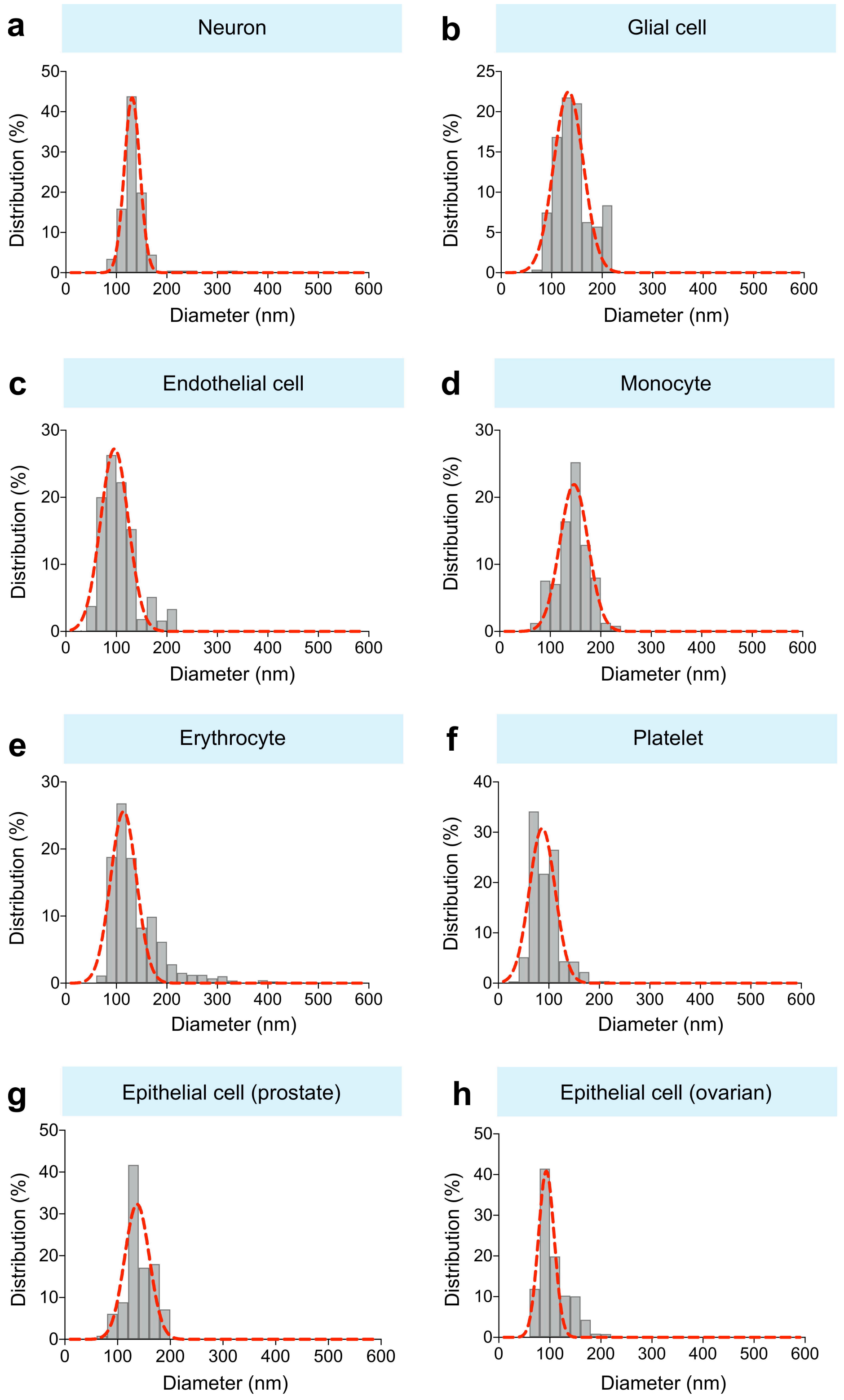

Supplementary Figure 11. Extracellular vesicles isolated from various cell origins. Extracellular vesicles were obtained from **(a)** neurons (SH-SY5Y), **(b)** glial cells (GLI36), **(c)** endothelial cells (HUVEC), **(d)** monocytes (THP-1), **(e)** erythrocytes, **(f)** platelets, **(g)** epithelial cells of prostate origin (PC-3), and **(h)** epithelial cells of ovarian origin (SK-OV-3), respectively. All vesicles were characterized with nanoparticle tracking analysis.

a

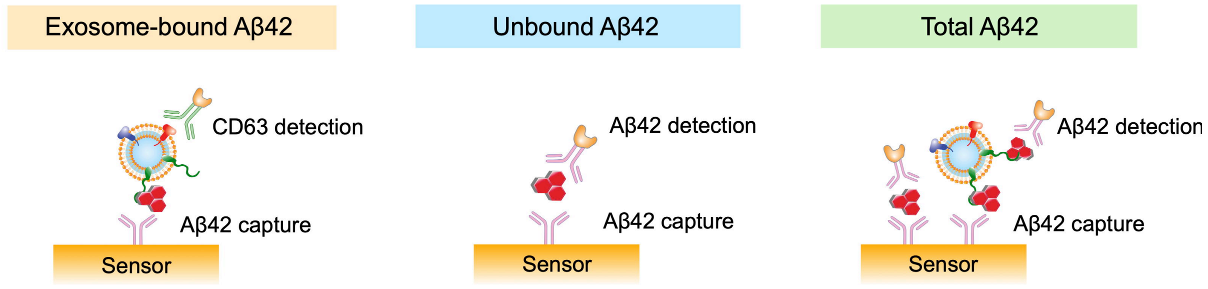

Assay	Exosome-bound Aβ42	Unbound Aβ42	Total Aβ42
Capture antibody	Aβ42	Aβ42	Aβ42
Detection antibody	CD63	Aβ42	Aβ42
Sample	Native plasma	Plasma filtrate (size cutoff = 50 nm)	Native plasma

b

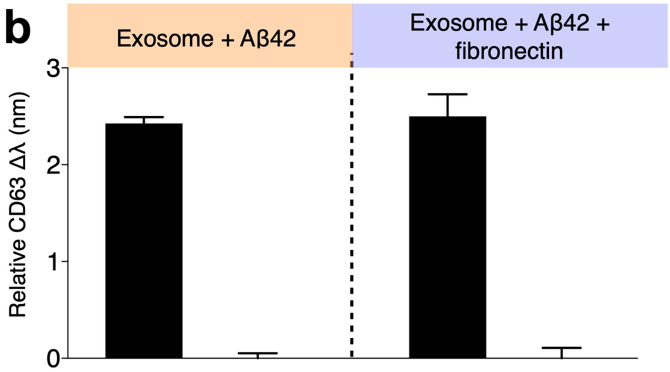

Capture antibody	Aβ42	IgG isotope control	Aβ42	IgG isotope control
Detection antibody	CD63	CD63	CD63	CD63

c

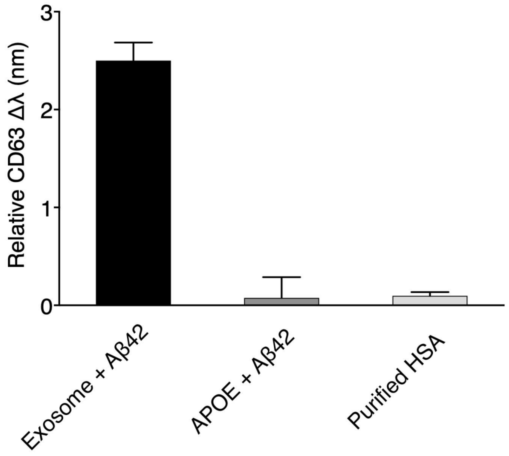

Supplementary Figure 12. APEX measurements of different populations of circulating Aβ. **(a)** APEX assay configurations for characterizing different populations of circulating Aβ in clinical plasma samples. Exosome-bound Aβ42 and total Aβ42 populations were measured from native plasma while unbound Aβ42 population was detected from plasma filtrate. **(b)** Incubation of fibronectin with exosome-bound Aβ42 resulted in negligible changes to the APEX signals. **(c)** Negative controls (APOE lipoprotein with Aβ42 protein, human serum albumin / HSA) showed negligible signals, demonstrating the APEX assay specificity for exosome-bound Aβ42. All measurements were performed in triplicate, and the data are displayed as mean ± s.d.

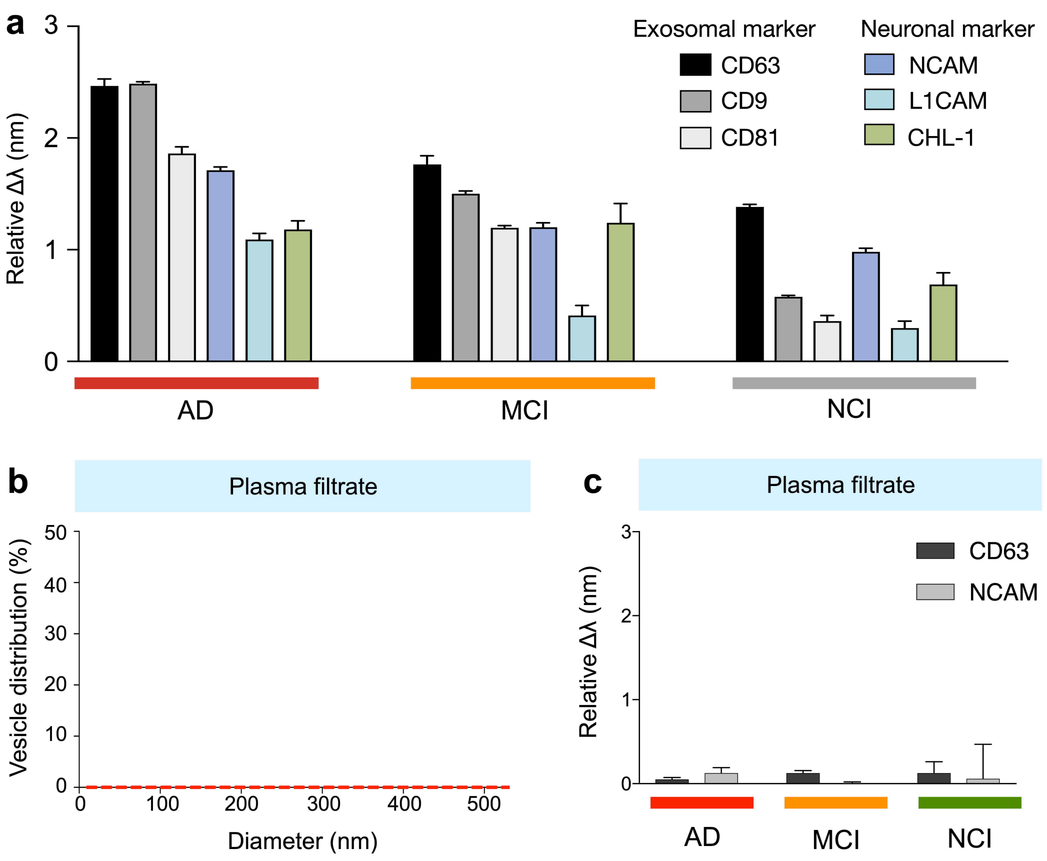

Supplementary Figure 13. Characterization of Aβ populations in clinical samples. (a) Exosome-bound Aβ42 population. We enriched for Aβ42 directly from native plasma samples, and measured the relative levels of co-localized signals for exosomal markers (CD63, CD81 and CD9) and neuronal markers (NCAM, L1CAM and CHL-1) in the captured Aβ42. All markers could be detected, with CD63 being the most highly expressed marker across the clinical samples tested. **(b)** Plasma filtrate for characterizing the unbound Aβ42 population. To evaluate the unbound Aβ42 population, we used membrane filtration (size cutoff = 50 nm, Nuclepore, Whatman) to prepare vesicle-free plasma filtrate. The filtrate showed negligible vesicle counts, as determined by nanoparticle tracking analysis. **(c)** The plasma filtrate also showed negligible signals for exosomal marker (CD63) and neuronal marker (NCAM), demonstrating the efficient removal of exosomes through filtration. Alzheimer's disease (AD), mild cognitive impairment (MCI), and healthy controls with no cognitive impairment (NCI). All measurements were performed in triplicate, and the data are displayed as mean ± s.d.

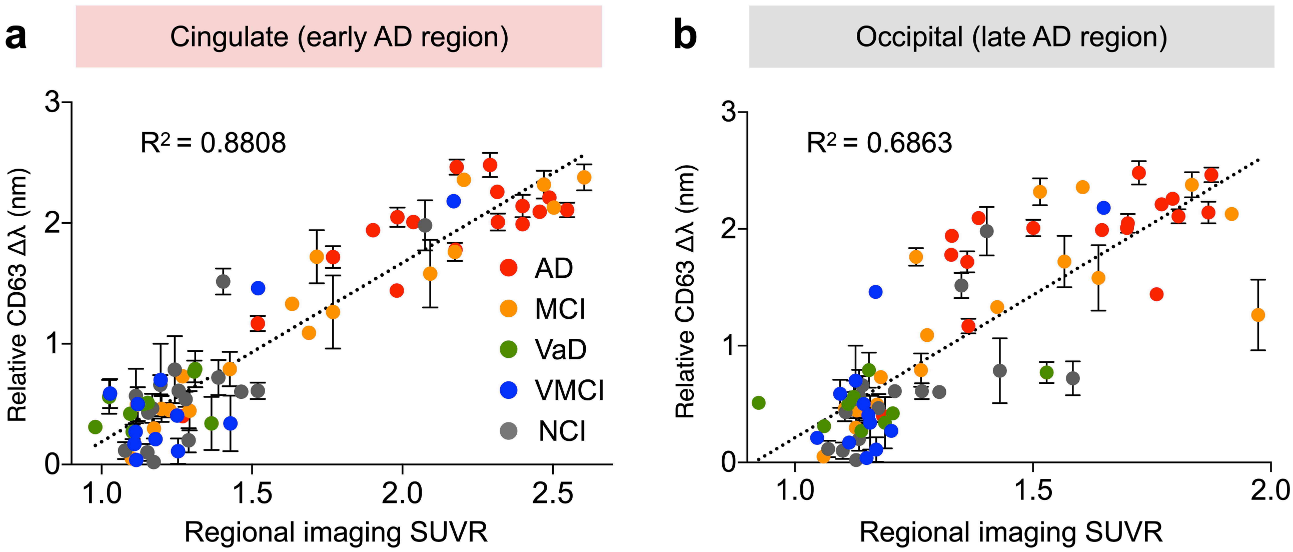

Supplementary Figure 14. Correlations of exosome-bound Aβ42 to regional brain amyloid load. We determined the imaging SUVR of specific brain regions, namely the early AD-affected cingulate region and the late AD-affected occipital region. APEX measurements of exosome-bound Aβ42 showed a better agreement to the imaging data of the early AD-affected region (**a**, R^2 = 0.8808) than to that of the late AD-affected region (**b**, R^2 = 0.6863). Alzheimer's disease (AD, n = 17), mild cognitive impairment (MCI, n = 18), vascular dementia (VaD, n = 9), vascular mild cognitive impairment (VMCI, n = 12), healthy controls with no cognitive impairment (NCI, n = 16). All measurements were performed in triplicate, and the data are displayed as mean ± s.d.

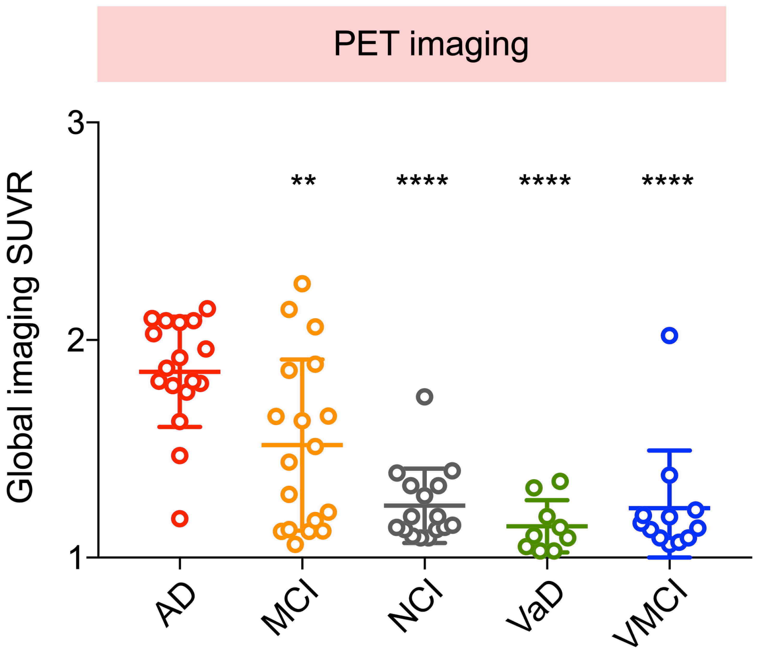

Supplementary Figure 15. Comparison of PET imaging in clinical subjects with different diagnoses. PET imaging of brain amyloid plaque burden was performed in patients with different clinical diagnoses ($n = 72$): AD ($n = 17$), MCI ($n = 18$), NCI ($n = 16$), VaD ($n = 9$) and VMCI ($n = 12$). Standardized Uptake Value Ratio (SUVR) of global average plaque deposition could distinguish between the AD clinical groups (AD and MCI), as well as from other healthy subjects (NCI) and clinical controls (VaD and VMCI) (**$P < 0.01$, ****$P < 0.0001$, Student's t-test).

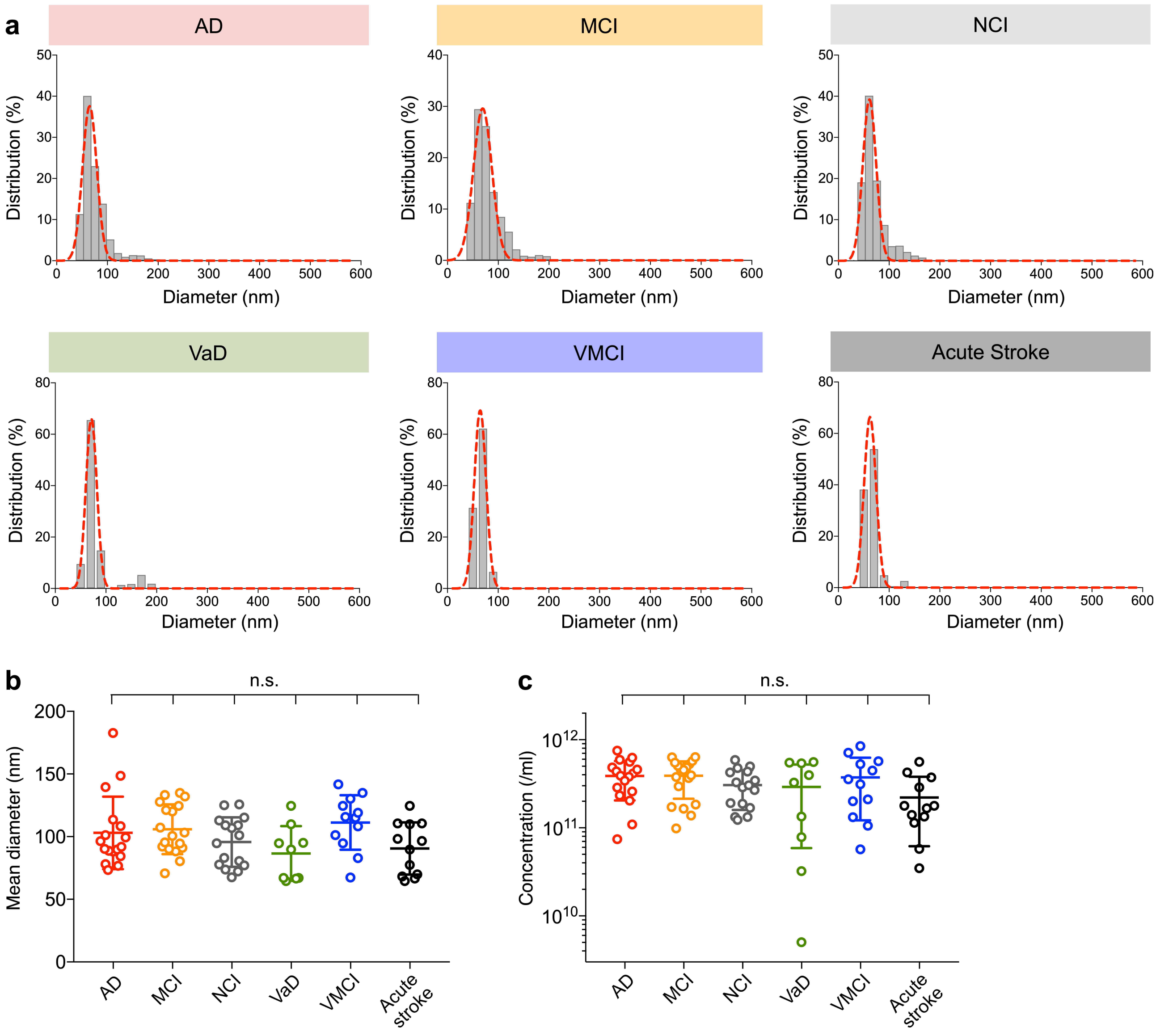

Supplementary Figure 16. Extracellular vesicles in clinical samples. (a) Representative analysis of extracellular vesicles, as measured by nanoparticle tracking analysis, of blood samples from subjects with different clinical diagnoses (AD = 17, MCI = 18, NCI = 16, VaD = 9, VMCI = 12, and acute stroke = 12). Comparisons of **(b)** vesicle size and **(c)** vesicle concentration from the clinical blood samples (*n* = 84). Note that neither vesicle size nor concentration was found to be significantly different among samples of different clinical diagnoses (n.s., not significant, Student's *t*-test).

Supplementary Table 1. APEX assay technology, sensor design and fabrication.

	APEX	Other SPR platforms
**Assay technology**	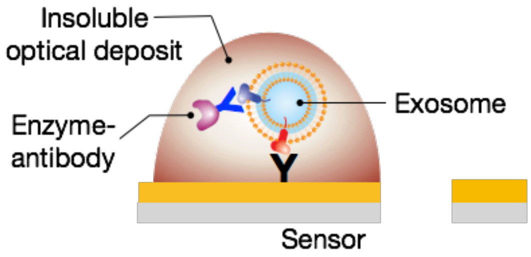 • Immuno-captures exosomes and enzymatically deposits a localized, insoluble optical product on sensor-bound exosomes<br>• Insoluble deposit forms only when multiple targets are co-localized in exosomes<br>• Insoluble deposit changes the refractive index and amplifies SPR detection signal<br>• Limit of detection ~ 200 exosomes	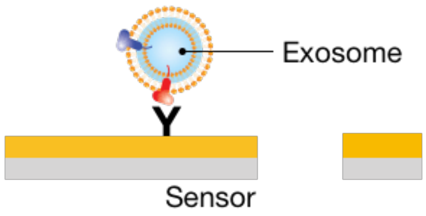 • Immuno-captures exosomes and detects via exosome-induced changes in refractive index[1-3]<br>• Limit of detection ~ 3000 exosomes[1]
**Possible targets**	• Detects diverse exosome proteins (extravesicular and intravesicular) and RNA targets<br>• Detects exosomal target co-localization (e.g., Aβ42+ CD63+)	• Detects exosome extravesciular proteins[1-3]<br>• Cannot detect target co-localization[1-3]
**Device design**	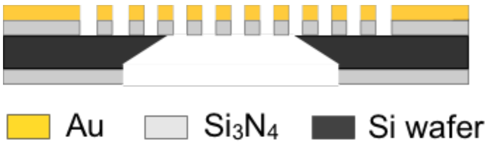 • Gold (Au) nanoholes suspended on a patterned silicon nitride (Si_3N_4) membrane<br>• Double-layered nanostructure	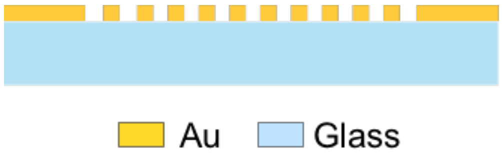 • Gold (Au) nanoholes on a uniform glass substrate<br>• Gold-on-glass[1]
**Optical Performance**	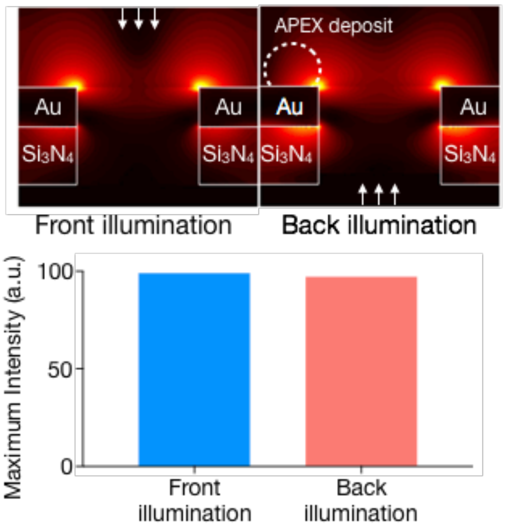	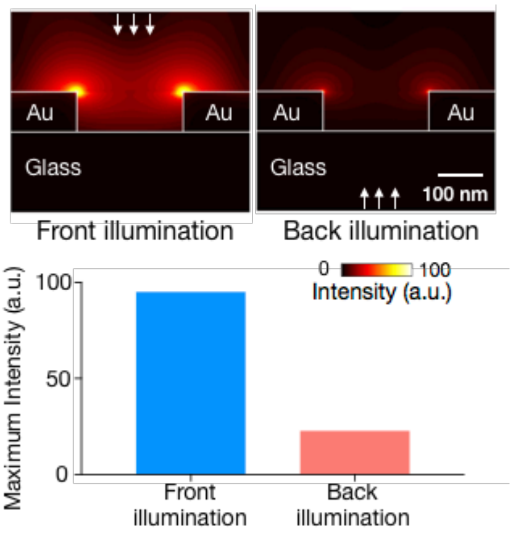

**Optical performance**	• APEX design enables bidirectional light excitation, likely due to Fano resonance in the coupled, double-layered plasmonic structure (i.e., periodic nanoholes in both Au and Si_3N_4 layers)[4-6]<br>• SPR can be excited by both front illumination (light illuminating from Au side) or back illumination (from Si_3N_4 side), and equally strong transmission intensity can be detected in both cases<br>• Back illumination minimizes direct incident illumination on the enzymatic APEX amplification (deposits on the Au side) and improves the analytical stability	• Gold-on-glass nanohole design supports only front illumination (light illuminating from Au side)
**Fabrication**	Deep ultraviolet lithography<br>• Advanced, parallel processing for precise nanohole pattern transfer<br>• Large, wafer-scale fabrication<br>• Compatible with well-established manufacturing process for mass production	Focused ion-beam milling<br>• Serial processing whereby every nanohole is sequentially milled<br>• Lengthy and costly process and thus only suitable for small-scale prototyping

Supplementary Table 2. List of markers and their probes used in profiling.

Protein	Description	Antibody, catalog no.
Aβ42	Amyloid-beta 42, a hydrophobic 42-peptide fragment from sequential cleavages by β- and γ-secretase of amyloid precursor protein.	Invitrogen, 374200; Abcam, ab34376
APP	Amyloid precursor protein, a large membrane protein that plays an essential role in neural growth and repair. It is enriched in synapses of neurons.	Fitzgerald,10R-1097; Biolegend 806001
α-syn	Alpha-synuclein protein, abundant in brain and localizes at the presynaptic nerve terminals.	Invitrogen, 32-8100; R&D Systems, AF1338
CD9	A tetraspanin scaffold glycoprotein that is abundant and characteristic in exosomes.	BD Biosciences, 555370
CD63	A type III lysosomal membrane protein, a member of the tetraspanin family, abundant and characteristic in exosomes.	BD Biosciences, 556019
CD81	Also known as TAPA-1, a widely expressed protein in the tetraspanin family known to associate with integrins and is characteristic in exosomes.	BD Biosciences, 555675
CHL1	Close homolog of L1, also known as neural cell adhesion molecule L1-like. A neural extracellular matrix and cell adhesion protein that plays a role in nervous system development and in synaptic plasticity and may be involved in signal transduction pathways.	R&D Systems, MAB2126, AF2126
IRS-1	Insulin receptor substrate 1, a signaling adapter protein which can mediate the control of various cellular processes by insulin.	R&D Systems, MAB39781; Fitzgerald, 70R-35306
L1CAM	Neural cell adhesion molecule L1. A transmembrane protein involved in processes such as neuronal migration, axonal growth, synaptogenesis, myelination and neuronal differentiation.	Invitrogen, 13-1719-82
NCAM	Neural cell adhesion molecule, a homophilic binding glycoprotein that is a cell adhesion molecule involved in neuron-neuron adhesion, neurite fasciculation and outgrowth of neurites. It plays a role in synaptic plasticity as well as learning and memory.	BD Biosciences, 559049; R&D Systems, AF2408
Tau	Tau, a microtubule-associated protein that stabilizes microtubules. It is abundant in neurons of the central nervous system.	Research Instruments, MN1000; R&D Systems, AF3494

Supplementary Table 3. Clinical information and PET imaging standardized uptake value ratio (SUVR).

Recruitment diagnosis	Number of subjects *n* = 84	Female (%)	Male (%)	Age Range	Median Age	Standardized uptake value ratio (SUVR)				
						Left-posterior-cingulate	Right-posterior-cingulate	Left-occipital	Right-occipital	Global average
Alzheimer's disease (AD)	17	14 (82%)	3 (18%)	57 - 89	76	1.26 - 2.58	1.28 - 2.51	1.17 - 1.94	1.20 - 1.97	1.18 - 2.14
Mild cognitive impairment (MCI)	18	7 (39%)	11 (61%)	58 - 82	75	1.05 - 2.66	1.11 - 2.54	1.07 - 1.84	1.05 - 2.28	1.06 - 2.26
No cognitive impairment (NCI)	16	7 (44%)	9 (56%)	55 - 83	75	1.12 - 1.97	1.02 - 2.18	1.07 - 1.48	1.07 - 1.73	1.09 - 1.74
Vascular dementia (VaD)	9	5 (56%)	4 (44%)	59 - 91	76	0.93 - 1.41	0.90 - 1.32	0.92 - 1.54	0.92 - 1.52	1.03 - 1.35
Vascular mild cognitive impairment (VMCI)	12	7 (58%)	5 (42%)	62 - 81	72	1.04 - 2.20	1.01 - 2.14	1.04 - 1.65	1.05 - 1.65	1.06 - 2.02
Acute stroke	12	Anonymous and de-identified				PET imaging of amyloid plaque not performed				

AD: Alzheimer's disease, MCI: mild cognitive impairment, NCI: no cognitive impairment, VaD: vascular dementia, VMCI: vascular mild cognitive impairment.

Supplementary References

1. Im, H. et al. Label-free detection and molecular profiling of exosomes with a nano-plasmonic sensor. *Nat Biotechnol* **32**, 490-495 (2014).
2. Zhu, L. et al. Label-free quantitative detection of tumor-derived exosomes through surface plasmon resonance imaging. *Anal Chem* **86**, 8857-8864 (2014).
3. Liu, C. et al. Sensitive detection of exosomal proteins via a compact surface plasmon resonance biosensor for cancer diagnosis. *ACS Sensors* **3**, 1471-1479 (2018).
4. Yanik, A. A. et al. Seeing protein monolayers with naked eye through plasmonic Fano resonances. *Proc Natl Acad Sci U S A* **108**, 11784-11789 (2011).
5. Dana, B. & Bahabad, A. Double Fano resonance in a plasmonic double grating structure. *Opt Express* **24**, 22334-22344 (2016).
6. Luk'yanchuk, B. et al. The Fano resonance in plasmonic nanostructures and metamaterials. *Nat Mater* **9**, 707-715 (2010).
