# Supplementary material for: Subtyping of circulating exosome-bound amyloid β reflects brain plaque deposition
Source: Nat Commun. 2019 Mar 8;10:1144. doi: 10.1038/s41467-019-09030-2 (PMC6408581; doi:10.1038/s41467-019-09030-2)
Supplement: Supplementary file 2 — Reporting Summary Checklist [file 41467_2019_9030_MOESM2_ESM.pdf]

nature research

Corresponding author(s): Huilin SHAO (NCOMMS-18-30579B)

Reporting Summary

Nature Research wishes to improve the reproducibility of the work that we publish. This form provides structure for consistency and transparency in reporting. For further information on Nature Research policies, see Authors & Referees and the Editorial Policy Checklist.

Statistical parameters

When statistical analyses are reported, confirm that the following items are present in the relevant location (e.g. figure legend, table legend, main text, or Methods section).

n/a	Confirmed	
☐	☒	The exact sample size (*n*) for each experimental group/condition, given as a discrete number and unit of measurement
☐	☒	An indication of whether measurements were taken from distinct samples or whether the same sample was measured repeatedly
☐	☒	The statistical test(s) used AND whether they are one- or two-sided<br>*Only common tests should be described solely by name; describe more complex techniques in the Methods section.*
☐	☒	A description of all covariates tested
☐	☒	A description of any assumptions or corrections, such as tests of normality and adjustment for multiple comparisons
☐	☒	A full description of the statistics including central tendency (e.g. means) or other basic estimates (e.g. regression coefficient) AND variation (e.g. standard deviation) or associated estimates of uncertainty (e.g. confidence intervals)
☐	☒	For null hypothesis testing, the test statistic (e.g. *F*, *t*, *r*) with confidence intervals, effect sizes, degrees of freedom and *P* value noted<br>*Give P values as exact values whenever suitable.*
☒	☐	For Bayesian analysis, information on the choice of priors and Markov chain Monte Carlo settings
☒	☐	For hierarchical and complex designs, identification of the appropriate level for tests and full reporting of outcomes
☐	☒	Estimates of effect sizes (e.g. Cohen's *d*, Pearson's *r*), indicating how they were calculated
☐	☒	Clearly defined error bars<br>*State explicitly what error bars represent (e.g. SD, SE, CI)*

Our web collection on statistics for biologists may be useful.

Software and code

Policy information about availability of computer code

Data collection	OceanView (real-time spectral acquisition and analysis).
Data analysis	R-package (version 3.4.2) and Graphpad Prism 7.

For manuscripts utilizing custom algorithms or software that are central to the research but not yet described in published literature, software must be made available to editors/reviewers upon request. We strongly encourage code deposition in a community repository (e.g. GitHub). See the Nature Research guidelines for submitting code & software for further information.

Data

Policy information about availability of data

All manuscripts must include a data availability statement. This statement should provide the following information, where applicable:

- Accession codes, unique identifiers, or web links for publicly available datasets
- A list of figures that have associated raw data
- A description of any restrictions on data availability

Data supporting the findings of this study are available from the corresponding author on reasonable request.

Field-specific reporting

Please select the best fit for your research. If you are not sure, read the appropriate sections before making your selection.

☒ Life sciences ☐ Behavioural & social sciences ☐ Ecological, evolutionary & environmental sciences

For a reference copy of the document with all sections, see nature.com/authors/policies/ReportingSummary-flat.pdf

Life sciences study design

All studies must disclose on these points even when the disclosure is negative.

Sample size	n = 84 (17 AD patients, 18 mild cognitive impairment, 16 no cognitive impairment, 9 vascular dementia, 12 vascular mild cognitive impairment, 12 acute stroke). All samples were age-matched. We used a comparable number of samples from each clinical group for correlational analysis of APEX blood measurements (exosome-bound, unbound and total circulating Ab) with brain plaque deposition. The correlation of determination (R2) was performed at 95% confidence interval for all samples.
Data exclusions	No data were excluded.
Replication	All measurements were performed as triplicates and all data were presented.
Randomization	NA.
Blinding	All experiments were performed blinded from clinical diagnoses and PET measurements.

Reporting for specific materials, systems and methods

Materials & experimental systems

n/a	Involved in the study
☒	☐ Unique biological materials
☐	☒ Antibodies
☐	☒ Eukaryotic cell lines
☒	☐ Palaeontology
☒	☐ Animals and other organisms
☐	☒ Human research participants

Methods

n/a	Involved in the study
☒	☐ ChIP-seq
☒	☐ Flow cytometry
☒	☐ MRI-based neuroimaging

Antibodies

Antibodies used	All antibodies and their catalog numbers are provided in Supplementary Table 2.
Validation	All validation statements are found on the manufacturers' websites.

Eukaryotic cell lines

Policy information about cell lines

Cell line source(s)	ATCC.
Authentication	ATCC authentication.
Mycoplasma contamination	All cell lines were tested negative for mycoplasma contamination.
Commonly misidentified lines (See ICLAC register)	NA.

Human research participants

Policy information about studies involving human research participants

Population characteristics	Patients were age and gender matched, and diagnosed through independent neuropsychological assessments.
Recruitment	No selection bias. Patients were recruited based on clinical diagnosis determined via independent neuropsychological assessments.
